# Toll-Like Signaling and the Cytokine IL-6 Regulate Histone Deacetylase Dependent Neuronal Survival

**DOI:** 10.1371/journal.pone.0041033

**Published:** 2012-07-27

**Authors:** Nicole Forgione, Vincent Tropepe

**Affiliations:** 1 Department of Cell and Systems Biology, University of Toronto, Toronto, Ontario, Canada; 2 Centre for the Analysis of Genome Evolution and Function, University of Toronto, Toronto, Ontario, Canada; Peking University Health Science Center, China

## Abstract

Histone deacetylase (HDAC) proteins have a role in promoting neuronal survival *in vitro*, but the mechanism underlying this function has not been identified. Here we provide evidence that components of the neuronal microenvironment, including non-neuronal cells and defined culture media, can mitigate midbrain neuronal cell death induced by HDAC inhibitor treatment. Using microarrays we further identified gene expression changes taking place in non-neuronal cells as a result of HDAC inhibition. This analysis demonstrated that HDAC inhibitor treatment results in the down-regulation of immunity related signaling factors, in particular the Toll-like receptors (TLR). TLR signaling is active in cultured midbrain cells, yet blocking TLR receptors is not sufficient to cause neuronal cell death. In contrast, selective activation of this pathway using TLR ligands can modestly block the effects of HDAC inhibition. Furthermore, we observed that the negative effects of HDAC inhibitor treatment on neuronal survival could be more substantially blocked by the cytokine Interleukin-6 (IL-6), which is a major downstream target of TLR signaling. These data suggest that HDACs function to promote neuronal survival by activating a TLR and IL-6 dependent pathway.

## Introduction

Neuron-astrocyte interactions play a key role in neuron survival and homeostasis. Astrocytes directly regulate synaptic transmission by releasing synaptically active molecules such as glutamate, purines, and γ-Aminobutyric acid (GABA) [1]. Neurons have limited antioxidant capacity and therefore rely on astrocytes for protection against oxidative stress [2], [3]. Astrocytes also play an indispensible role in glutamate, ion, and water homeostasis, which in turn regulates neuronal energy metabolism [4]. The synthesis and release of neurotrophic factors such as nerve growth factor (NGF), brain-derived neurotrophic factor (BDNF), ciliary neurotrophic factor (CNTF), and glial cell line-derived neurotrophic factor (GDNF) by astrocytes in response to injury contributes to neuronal survival [5]. Interestingly, astrocytes as well as microglia are also known to promote neuronal survival by mediating the neuroimmune response [6]. Thus, communication between cells is crucial, but the intracellular mechanisms that enable cells, in particular glial cells, to release growth or immune-related factors to promote neuronal survival are not well characterized.

We have previously demonstrated that the survival of midbrain neurons in a heterogeneous population of neuronal and glial cells is severely compromised after the function of histone deacetylases (HDACs) have been blocked by acute exposure to histone deacetylase inhibitors (HDACi) [7]. HDACs work in concert with histone acetyltransferases (HATs) to dynamically regulate the acetylation state of both histone and non-histone proteins. HDACs are expressed in all eukaryotic organisms, where they regulate core biological processes such cellular proliferation, differentiation and homeostasis. HDACs are expressed throughout the developing and adult mammalian nervous system [8]. HDAC1 and HDAC2 are expressed in a developmentally regulated manner in neurons and glia [9]. Conditional deletion of HDAC1 and HDAC2 in the mouse brain results in gross morphological abnormalities in the cortex, hippocampus and cerebellum. These defects result from disruptions in the proliferation and survival of neuronal progenitors [10]. The importance of HDACs in supporting neuronal function and homeostasis is highlighted by the implication of HDAC dysfunction in the development of neurodegenerative diseases [11]. While HDACs deacetylate a variety of non-histone proteins including α-tubulin and HSP-70, their main role is to regulate transcription via direct deacetylation of core histone proteins and interaction with co-repressors. Therefore it is likely that HDACs play a role in neuronal survival by regulating the transcription of key survival signals. However, whether these effects are direct (within neurons) or indirect (via non-neuronal cells, such as astrocytes) is not known. Given the important function of glial cells in promoting neuronal survival, we wanted to test if HDACi might be causing a defect in glial-dependent regulation of neuronal survival in a non-autonomous manner, possibly initiated by alterations in gene expression.

In order to identify factors expressed by non-neuronal cells that contribute to the effects of HDACi on neurons we carried out gene expression profiling of HDACi treated midbrain cultures composed mainly of astrocytes. This analysis showed a strong down-regulation of innate immune-related factors such as chemokines, cytokines, and pattern recognition receptors, supporting the possibility that astrocytes influence neuronal survival through the expression of neuroimmune factors. While microglia are the main modulators of immunity in the brain, astrocytes are also known to express similar neuroimmune factors [12], such as the pattern recognition Toll-like receptors (TLRs), that are responsible for detecting changes in the environment and triggering an innate immune response [13]. Our data also shows that TLR activation and the cytokine IL-6, a downstream target of TLR signaling, have a significant role in mediating HDAC dependent neuronal survival. Thus, expression of genes involved in signaling pathways normally required for innate immunity have a complementary function in mediating neuronal survival. Our data also suggests that astrocytes might provide these neuronal survival cues in a non-autonomous manner.

## Methods

### Ethics Statement

Housing and breeding of CD1 Mice (Charles River Laboratories; St. Constant, Quebec) was performed in accordance with the regulations on animal experimentation established by the Canadian Council on Animal Care. The experimental procedures were approved by the University of Toronto Animal Care Committee.

### Cell Culture and Drug Treatments

Primary cells were obtained from the ventral midbrain tissue of embryonic day (E) 14.0–15.0 CD1 mice, collected in cold PBS and triturated to yield a single cell suspension. 5×10^5^ cells/well were plated on chambered glass slides (BD Falcon) coated with 0.5% laminin and 0.5% fibronectin (Sigma). Cells were grown in DMEM with 10% FBS for 14 days. Primary cortical cells were obtained from E12.5–13.5 embryos. Stock solutions for trichostatin A (TSA) (Upstate-Millipore), sodium butyrate (SB), (Upstate-Millipore) and valproic acid (VPA) (Calbiochem) were prepared according to the manufacturer’s instructions. Freshly prepared media containing specific inhibitors were used according to previously established concentrations [7]. Lipopolysaccharide (LPS) was obtained from Sigma and used at a final concentration of 100 ng/ml. The TLR4 inhibitor CLI-095 was used at a final concentration of 3 µM. Oxidated 1-palmitoyl-2-archidonyl-sn-glycero-3-phosphorylcholine (OxPAPC) was used to treat cells at a concentration of 30 µg/ml. Chloroquine was used at a final concentration of 30 µM. Poly I:C was used at a concentration of 10 µg/ml. Recombinant IL-6 (Millipore) was used to treat midbrain cultures at a concentration of 2 ng/ml.

### Immunocytochemistry

Cells were fixed in buffered 4% paraformaldehyde for 30 minutes at room temperature followed by three washes in PBS. After fixation cells were permeabilized by incubating with 0.3% Triton X-100 for 5 minutes. Primary antibodies were diluted in PBT with 10% normal goat serum and incubated with the cells for 2 hours at 37°C. Following incubation with primary antibodies, cells were washed three times in PBS. Secondary antibodies were diluted in PBS with 2% normal goat serum and incubated with the cells for 30 minutes at 37°C. All secondary antibodies were used at a dilution of 1∶100 and obtained from Jackson Immunoreasearch. Following incubation with secondary antibodies, cells were washed an additional three times in PBS. Nuclear staining was performed by mounting samples using ProLong Gold Anti-Fade Mountant containing DAPI (Invitrogen). Anti-βIII tubulin (TU-20; Abcam) was used at 1∶100. Ionized calcium binding adaptor molecule 1 (Iba1) (Wako Chemicals) was used at a concentration of 1 µg/ml. TLR4 antibody staining was carried out in the absence of Triton X-100 to maintain the integrity of cell membranes. TLR4 antibody was used at a concentration of 1∶100 and was obtained from Abcam. For all antibodies a no primary antibody control was carried to rule out non-specific signal.

### Imaging, Cell Counting and Statistics

Confocal microscopy was performed using the Leica TCS SP5. Cell counts were performed on immunostained cells using Volocity analysis software (Improvision). First a Z-stack was obtained using the 20X objective, for three separate areas in an individual well. The total number of nuclei (labeled with DAPI) and immunolabeled cells in the maximum projection of each Z-stack was counted using Volocity analysis software. The size of each area counted (i.e. the size of the image) was equal to 775 µM × 775 µM. The surface area of the culture well was equal to 0.7 cm^2^. Therefore, one measurement area was equal to 0.1% of the total area of each well. The total number of cells in an individual culture was calculated by determining the average number of cells in 3 separate fields of view. The number of immunolabeled cells is always represented as a percentage of the total number of DAPI labeled cells. Using this method, cell counts were obtained by first averaging experimental replicates and then averaging across biological replicates. Either a Student’s t-test for pair-wise comparisons of the means between treatment groups, or a one-way ANOVA followed by a Tukey-Kramer MSD test for pair-wise significant differences between treatment groups was used with a threshold of p<0.05 for statistical significance. Statistical tests were performed using Microsoft Excel 2008.

### Microarray Expression Profiling

Microarray expression profiling was performed at the University Health Network-Microarray Centre (UHN-MAC, www.microarrays.ca). Quality-validated RNA was amplified and then reversed transcribed into cDNA using Agilent’s Low RNA Input Amplification kit. The cDNA was labeled with (single-colour) Cy3 fluorescent probes and hybridized to Agilent's inkjet *in situ* synthesized 60-mer DNA oligonucleotide microarrays (Whole Mouse Genome expression microarray chips, 4 × 44 K, version G4122F). DNA microarray chips were then scanned with the SureScan technology (Agilent).

### Microarray Data Normalization and Statistical Analysis

Three biological replicates were performed for each of the 3 inhibitory optimized HDACi treatments (330 nM TSA, 50 mM SB and 50 mM VPA) and the untreated control. A total of 12 hybridizations were performed on 4 microarray slides. The background subtraction, expression summary, and normalization of gene signals were carried out using the visualization and analysis software GeneSpring GX. Z-tests were performed using an EXCEL template (MicroSoft Inc.). Briefly, the “gProcessesSignal” values of Agilent output files were used as the raw data. Log base 10 transformations were applied to the raw data. A grand average of all arrays was also obtained and used to normalize each array. The Z-score was then calculated by standardization (with subtraction of the average and subsequently divided by the standard deviation, SD, of each array). Changes (Z-Test) due to the drug treatments were calculated for each gene according to the following formula:
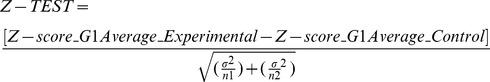
where G1 represents the average Z-score for any particular gene being tested under multiple experimental conditions (in this case, experimental versus control). The mean difference is corrected by the standard error (SE) for the difference between means where 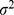 is the SD of repeated hybridization intensity measurements (expressed as Z scores) for either condition 1 or condition 2, and n equals the number of repeated measurements for either condition 1 or condition 2. P-values are estimated by a percentile ranking. Finally, standardization was applied to the Z-test statistics. Genes at the top and bottom 1% percentile (i.e. p-value <0.02) were considered statistically significant and termed differentially expressed genes (DEG). A Venn Diagram of DEGs compared between drug treatment groups and control was prepared to identify common and uniquely active genes. The common subset of DEGs in all treatments were subjected to Exploratory Data Analysis (EDA) using the web database GENECOIDS.

### RT-qPCR Validation of Microarray Results

A subset of 6 DEGs determined by the microarray analysis was validated by RT-qPCR. StrataScript RT enzyme (Stratagene) was used to synthesize first strand cDNAs from 1 mg of total RNA, using an oligo(dT_21_) primer to anneal to mRNAs. cDNA was diluted to 1 ng/ml to be used in qPCR experiments. Each 25 µl reaction contained 7.25 µl of DEPC-treated water, 2.5 µl of 10X PCR buffer (200 mM Tris-HCl, pH 8.4; 200 mM KCl, Tween-20) (BioShop), 2.0 µl of 25 mM MgCl_2_, 2 µl of dNTP mixture (5 mM of each), 4 µl of 50% glycerol, 0.375 µl of 2 mM ROX reference dye (Stratagene), 0.75 µl of 5 mM Sybr Green in DMSO (Cambrex), 0.5 µl of both forward and reverse primers (25 mM of each), 0.125 µl of 5U/ml Taq DNA polymerase (BioShop) and 5 µl of 1 ng/ml cDNA template. For each gene studied, two control reactions were included: an RNA sample from a cDNA synthesis without RT enzyme (NoRT) and a control reaction without cDNA template (NTC). For the No-RT controls, 5 µl of 1 ng/ml of RNA were used. For the NTC, 5 µl of DEPC-treated water were added instead of cDNA template. The real-time PCR reactions were performed on the Mx3000P instrument (Stratagene) as follows: one cycle at 95°C for 10 min; 40 cycles at 95°C for 30 seconds, 53°C (group 1 primers) or 56°C (Group 2 primers) for 1 minute, and 72°C for 1 minute. A standard curve was constructed for every gene, and the efficiency of PCR amplification was calculated from the slope of the plot (% efficiency = 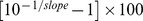). A melting curve analysis of the amplified product was performed after the PCR reaction for each gene to detect the presence of any primer dimers. Each reaction was performed in triplicate. Primer sequences and amplicon sizes can be found in Table 1.

**Table 1:** qPCR primer sequences and expected amplicon size.

Gene Name	Forward Primer	Reverse Primer	Amplicon (bp)
GAPDH	GGTGCTGAGTATGTCGTGGA	CCTTCCACAATGCCAAAGTT	284
GFAP	ACAAATCACTTCCTTCATCC	TAGAGAGACTTTGCCTCAGG	125
Nefm	GTGAAGTCTCCTGAGGCTAA	TCTGCCTGGTCTGACTTGAC	113
Hoxa10	CCAGCCCTGGGTAAACTTAGC	CGGCTCCTTGCACCATTG	87
TLR4	AATACAAGCCATGTCATGTT	GGAATGAAGACCTCTCAAAA	140
Robo3	GAATCGCCGAGAGGAACCAA	ACATCGGTTGACCAGGGAAG	100
Nurr1	CCTGTCAGCACTACGGTGTT	CTTGTCCACTGGGCAGTTTT	116

### Semi-Quantitative RT-PCR

Total RNA was obtained from differentiated cell cultures using the Purelink Micro-to-Midi Total RNA Purification kit from Invitrogen and cDNA was synthesized using SuperScript First Stand synthesis system (Invitrogen). Primers for β-actin were used for linear range amplification of cDNA template in a standard PCR reaction to establish normalized template concentration and amplified products were resolved on a 1% agarose gel containing ethidium bromide by electrophoresis. Primer sequences and amplicon sizes can be found in Table 2. Each RNA sample was used in a cDNA synthesis reaction containing no reverse transcriptase (-RT). These samples were then amplified in a standard PCR reaction using β-actin as a control for genomic DNA contamination. Primer sequences and amplicon sizes can be found in Table 2. A no template control was carried for each primer set to rule out contamination of the PCR reaction.

**Table 2:** Semi-qPCR primer sequences and expected amplicon size.

Gene Name	Forward Primer	Reverse Primer	Amplicon (bp)
β-Actin	TGTTACCCAACTGGGACGACA	TCTCAGCTGTGGTGGTGAAG	392
TNFα	GAACTGGCAGAAGAGGCACT	AGGGTCTGGGCCATAGAACT	203
IL-6	AGTTGCCTTCTTGGGACTGA	TCCACGATTTCCCAGAGAAC	159
IL-1β	GCCCATCCTCTGTGACTCAT	AGGCCACAGGTATTTTGTCG	230

### Chromatin Immunoprecipitation (ChIP) - qPCR

Cells obtained from E14 midbrains were cultured for 2 weeks under differentiation conditions at a density of 500 cells/µl. In order to facilitate the collection of the appropriate number of cells for the desired number of immunoprecipitations, cells were cultured in 10 cm dishes. After the two-week culture period, one group of cells was treated with 50 mM SB for 48 hours, and the other group of cells serving as a control, did not receive any drug treatment. Two biological replicates of this experiment were carried out. For each replicate, primary embryonic midbrain tissue was obtained from litters from different females. ChIP was carried out according to the protocol specified by the EZ-ChIP Chromatin Immunoprecipitation Kit (Millipore). Cells were fixed using a final concentration of 1% PFA for 10 minutes. Un-reacted PFA was quenched using glycine, and cells were collected using a plastic cell scraper. Cell lysates were sonicated, and underwent gel electrophoresis to ensure that DNA was sheared into 100–1000 bp fragments. Following sonication overnight immunoprecipitations were carried out using Normal Mouse IgG (negative control) and anti-acetylated histone 3 lysine 9 (Millipore). DNA-protein complexes were eluted, cross-links were reversed, and DNA was purified. Amounts of DNA were analyzed using gel electrophoresis and quantitative PCR. Primers for qPCR can be found in Table 3. Real-time PCR reactions were performed using a Rotor Gene RG-3000 (Corbett Research) instrument. The following conditions were used for amplification: one cycle at 95°C for 5 minutes; 40 cycles at 95°C for 10 seconds, 59°C for 15 seconds, and 72°C for 25 seconds. A standard curve was generated for each gene using serial dilutions of a DNA template. The standard curve allowed for the determination of the efficiency of PCR amplification. A melting curve analysis was carried out for each amplified product to ensure that primers were specifically amplifying the gene of interest. Each reaction was performed in duplicate. Primary midbrain cells were obtained from E14 embryos and 3 biological replicates were performed, with each replicate consisting of primary cells obtained from embryos from different pregnant females. Primary cells were plated in 6 well plates coated with laminin and fibronectin. Cells were grown under differentiation conditions (10% FBS) for 14 days, with media changes every 3–5 days. Differentiated midbrain cultures were treated with 50 mM SB for 24 hours, and RNA was collected using the PureLink Micro-to-Midi Total RNA Purfication Kit (Invitrogen). SuperScript III Reverse Transcriptase (Invitrogen) was used to synthesize first strand cDNA from 3 µg of total RNA, using an oligo(dT_21_) primer to anneal mRNAs. All cDNAs were amplified by quantitative PCR in a 10 µl reaction consisting of 2 µl template, 2 µl of forward and reverse primer mix, 1 µl of PCR grade water, and 5 µl of LightCycler 480 SYBR Green I Master (Roche). For each gene studied, two controls were included: 1) a sample containing RNA without RT enzyme (NoRT), and 2) a no temple control reaction where template was replaced with PCR grade water. Real-time PCR reactions were performed on a Rotor Gene RG-3000 (Corbett Research) using the following conditions for amplification: one cycle at 95°C for 5 minutes; 40 cycles at 95°C for 10 seconds, 59°C for 15 seconds, and 72°C for 25 seconds. A standard curve was generated for each gene using serial dilutions of a cDNA template. The standard curve allowed for the determination of the efficiency of PCR amplification. A melting curve analysis was carried out for each amplified product to ensure that primers were specifically amplifying the gene of interest. Each reaction was performed in duplicate. Fold change in TLR4 expression in SB treated compared to control cultures was calculated using the delta delta Ct method:
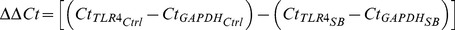



**Table 3:** ChIP-qPCR primer sequences and expected amplicon size.

Gene Name	Forward Primer	Reverse Primer	Amplicon (bp)
GAPDH	CCTTAGCCCTGAGCTGTGTC	ATGTTTTCTGGGGTGCAAAG	149
TLR4 (Primer #1)	GAACTGCAGAAGGCACTCAA	ATCAGTTGCCGTGTCTTGTG	176
TLR4 (Primer #2)	CCAGCTTCCTCTTGCTGTTC	GGAAGTGAGAGTGCCAACCT	90
ATF3 (Primer #1)	CCACGAGGGGACTGTTTTTA	CGGTCTGTTTTGCTGAGTGA	122
ATF3 (Primer #2)	CGGGAGCGTAGAGATCAAAG	GAACAGGGAATTCTCCACGA	147

Delta Delta Ct value was converted to fold change using the following equation: 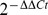.

The resulting fold change value was represented as log2 fold change.

## Results

### The Influence of the Microenvironment on the Survival of Midbrain Neurons

Our previous work has shown that HDACi treatment of midbrain cultures results in a significant loss of neurons, whereas astrocytes are relatively spared [7]. In contrast, similar HDACi treatment on cortical cultures had no effect on neuronal or glial survival in the same study. It is possible that cortical neurons are inherently resistant to the three different HDACi used (TSA, SB, and VPA), whereas midbrain neurons are more susceptible. Others have shown that relatively high concentrations of TSA (double what we used in our experiments) causes only minor, but reproducible cortical neuron cell death [14], supporting the notion that midbrain neurons might simply be less tolerant of HDACi at the concentrations we used. However, it is also possible that the differences in neuronal susceptibility due to HDACi treatment are related to differences in the non-neuronal (e.g. glial) cells in the population. For example, we noticed that cortical astrocytes had a distinct morphology compared to midbrain astrocytes. Moreover, optimal culture media conditions for maintaining neurons are known to be different for different brain tissues and as such the media conditions may also have a differential role in neuronal survival. These observations lead to the hypothesis that differences in the glial cell component of the microenvironment, or the media used, may be partly responsible for the different effects of HDACi treatment on midbrain versus cortical neurons.

**Figure 1:**
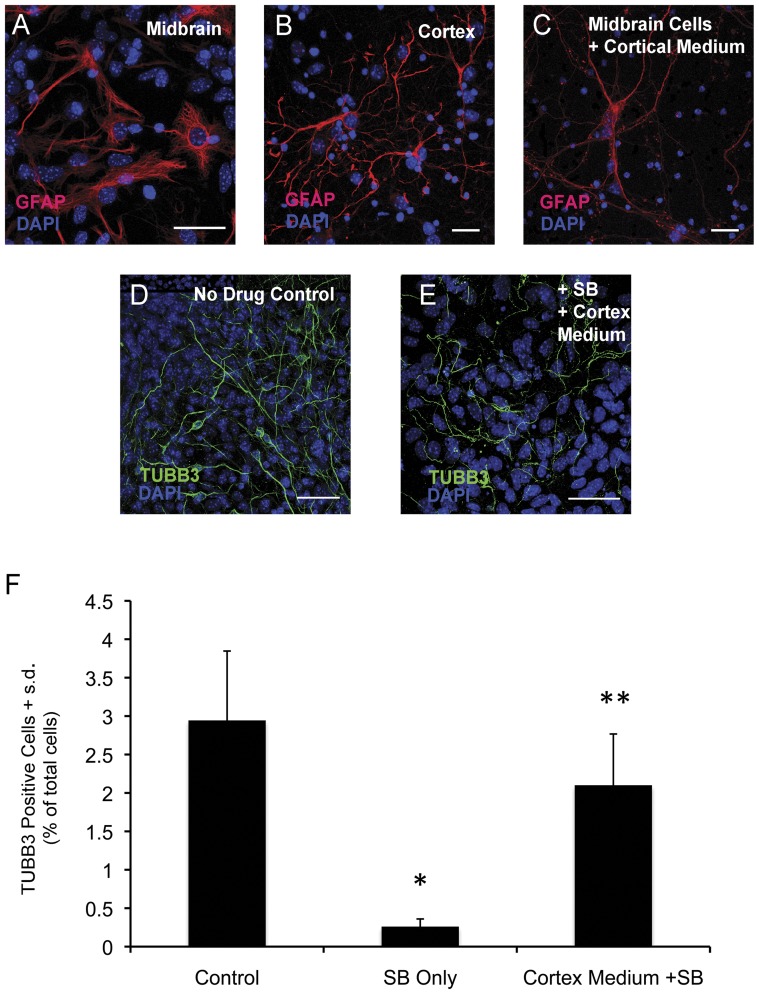
Microenvironmental factors in midbrain versus cortical cultures. (A) Protoplasmic GFAP positive astrocytes predominate in midbrain cultures. (B) GFAP positive astrocytes typical of cortical cultures, which display a filamentous morphology. (C) GFAP positive midbrain astrocyte cultured in cortical medium. Under cortical conditions midbrain derived astrocytes take on a filamentous morphology. (D) TUBB3 positive midbrain neurons cultured in 10% FBS for 14 days and then exposed to cortical medium alone for 48 hours. (E) TUBB3 positive midbrain neurons cultured in 10% FBS for 14 days and then treated with 50 mM SB in cortical medium for 48 hours. (F) Quantitation of the effect of different medium conditions on the TUBB3 positive neurons. Midbrain cells were cultured in 10% FBS for 14 days and then treated with 50 mM SB for 48 hours in the presence of cortical medium or 50 mM SB in 10% FBS. Bars represent the mean + s.d. (n = 5). Single asterisk (*) denotes treatment groups that are significantly different from the untreated control, as determined by an unpaired Student’s t-test (p<0.05). Double asterisks (**) indicate treatment groups that are significantly different from the SB only treatment group. Scale bars = 50 µM. Blue =  DAPI positive nuclei, red =  GFAP, green =  TUBB3.

Characterization of the morphological differences in GFAP expressing astrocytes in midbrain and cortical cultures revealed that midbrain astrocytes tend to have a protoplasmic appearance characterized by short, thick processes (Figure 1A), whereas GFAP positive astrocytes in cortical cultures can be described as filamentous, usually displaying long, thin processes (Figure 1B). The morphological differences between cultured midbrain and cortical astrocytes could be due to intrinsic differences in the differentiation of region-specific progenitor cells, or could be induced by the different cell culture environments (i.e. media components). To distinguish between these two possibilities, we cultured embryonic midbrain-derived cells in our standard cortical culture media and evaluated astrocyte morphology. When midbrain cells are cultured for two weeks in cortical medium, GFAP positive astrocytes take on a filamentous appearance (Figure 1C). Since conditioned medium from cortical cultures was not used, these data suggested that the media formulation itself has a direct impact on the morphological characteristics of astrocytes, which could in principle also influence the survival of neurons in response to HDACi. Based on these observations, we set out to further test whether cell non-autonomous factors can influence the responsiveness of midbrain neurons to HDACi treatment. We first asked whether cortical medium and its components confer a survival effect on midbrain neurons. Midbrain cells were cultured in 10% FBS for two weeks (standard midbrain differentiation conditions) and then treated with sodium butyrate (SB) in the presence of complete cortical medium (Figure 1D–F). Treating midbrain cultures with SB resulted in a >80% decrease in TUBB3+ neurons, consistent with our previous results [7]. In contrast, in treatment conditions that included cortical media in the presence of SB, the number of neurons was significantly increased compared to SB only treatment (Figure 1F). This suggests that complete cortical medium is able to protect midbrain neurons from HDACi-dependent cell death. However, the morphology of neurons in cultures treated with SB in the presence of cortical medium appears to be attenuated compared to control cultures (Figure 1D and 1E). Based on this observation we conclude that cortical medium can confer partial survival effects on neurons exposed to HDACi, however these conditions are not able to completely rescue the effects of HDAC inhibition, suggesting that there are other factors in cortical cultures, which contribute to the ability of cortical neurons to withstand HDACi treatment. One explanation for the incomplete rescue effect observed is that HDACi treatment decreases the production of important survival factors and that reintroducing them into the culture medium can partially block the deleterious effects of HDAC inhibition on midbrain neurons. We hypothesize that non-neuronal cells, such as astrocytes which make up ∼40% of the population of midbrain cells in our culture conditions, are a major source of these survival factors and that HDACi treatment might affect the ability of these cells to produce and/or secrete these factors.

### Gene Expression Profiling of HDAC Inhibitor Treated Midbrain Cultures

**Figure 2:**
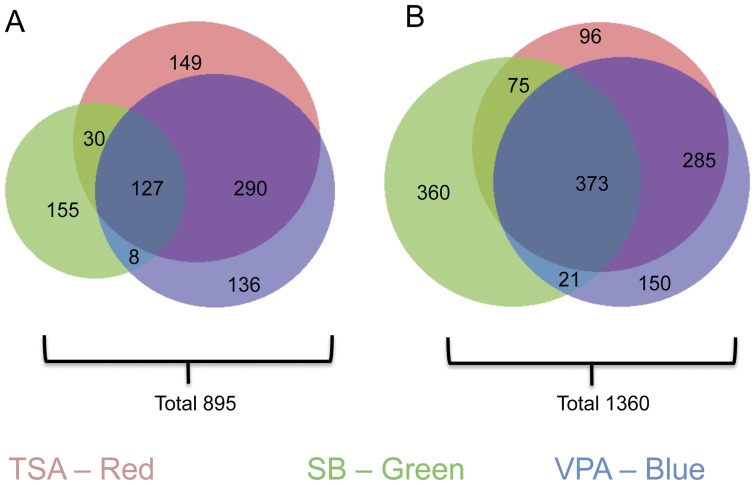
Venn analysis of gene expression changes associated with HDACi treatment of differentiated midbrain cultures. Differentiated midbrain cultures were treated with 330 nM TSA, 50 mM SB or 50 mM VPA respectively for 48 hours and gene expression was analyzed using Agilent whole mouse genome expression microarray chips. Genes with a p-value of <0.02 were considered differentially expressed (DEGs) and subjected to Venn analysis. (A) A total of 895 genes are up-regulated by HDACi treatment. A subset of 127 of these genes are up-regulated by all three drug. (B) A total of 1360 genes are down-regulated as a result of HDACi treatment, with a subset of 373 genes being down-regulated by all three drugs. Numbers represent the total number of modulated genes in that experimental condition.

To test for cell non-autonomous factors primarily derived from astrocytes that could potentially mediate HDAC dependent neuronal survival, we carried out gene expression profiling studies on HDACi treated midbrain cultures. In order to focus the analysis on gene expression changes primarily in astrocytes, we chose to perform gene expression profiling after 48 hours of drug treatment. At this stage, HDACi treatment has significantly reduced the numbers of neurons and proliferating progenitor cells and therefore viable cells in these midbrain cultures consist mainly of astrocytes [7]. Microarray analysis was carried out on differentiated primary midbrain cultured cells treated with 330 nM TSA, 50 mM SB, or 50 mM VPA for 48 hours compared to untreated control cultures. We found a total of 2255 differentially expressed genes (DEGs), which represented approximately 6.8% of the total gene set analyzed. This result is consistent with previous studies showing that HDAC inhibition generally results in the deregulation of relatively small subsets of genes. This trend is illustrated by gene expression profiling studies carried out in cancer cell lines, in which HDACi treatment resulted in the deregulation of approximately 2–7% of the total gene set analyzed [15], [16]. Similarly, HDACi treatment of rat cortical neurons deregulated the expression of 8% of the total gene set analyzed [17]. Of the 2255 DEGs identified from our analysis, 895 genes were up-regulated (Figure 2A) when compared to an untreated control and 1360 genes were down-regulated (Figure 2B). A Venn analysis of up- and down-regulated genes showed that all three drugs up-regulated a common subset of 127 genes and down-regulated a different common subset of 373 genes. A full list of the common subset of up-regulated or down-regulated genes can be found in Table S1 or Table S2, respectively. Since each of the three HDACi have a similar effect on promoting midbrain neuronal cell death, we reasoned that DEGs that were commonly observed between the three HDACi data sets more accurately reflected the overall specific effects of decreased HDAC activity in the cells, which are known to express several different HDAC genes [7], as opposed to the unique inhibition of specific HDACs by the different inhibitors or to off target effects.

**Figure 3:**
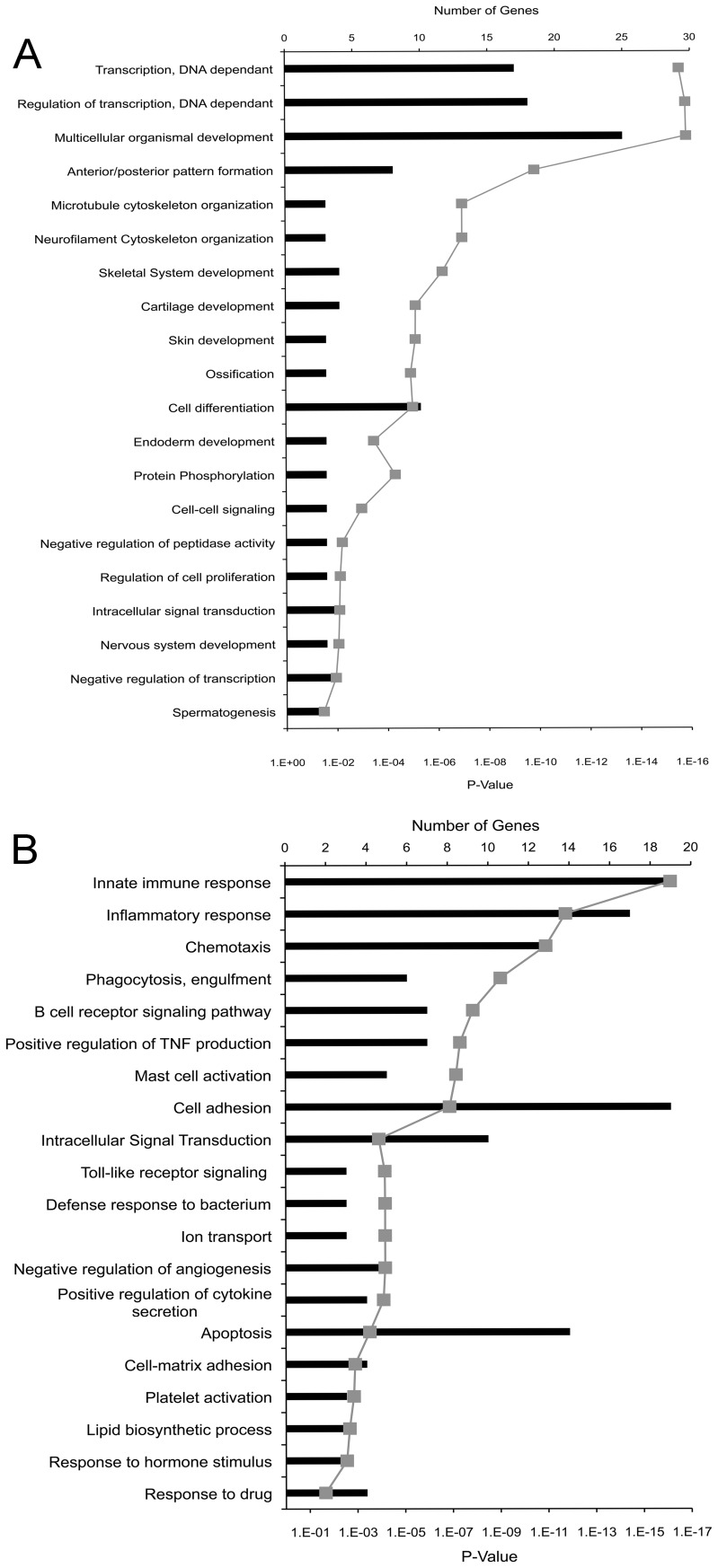
Go term enrichment analysis: General biological processes associated with HDACi-regulated genes. Gene Ontology (GO) term enrichment analysis was carried out using GENECODIS on (A) 127 genes that are up-regulated by all three drugs, and (B) 373 genes that are down-regulated by all three drugs. Black bars represent the number of genes annotated to the GO terms listed along the y-axis. Grey diamonds indicated the P-value, which represents the probability that an individual gene is associated to a particular GO term based on chance alone.

**Figure 4:**
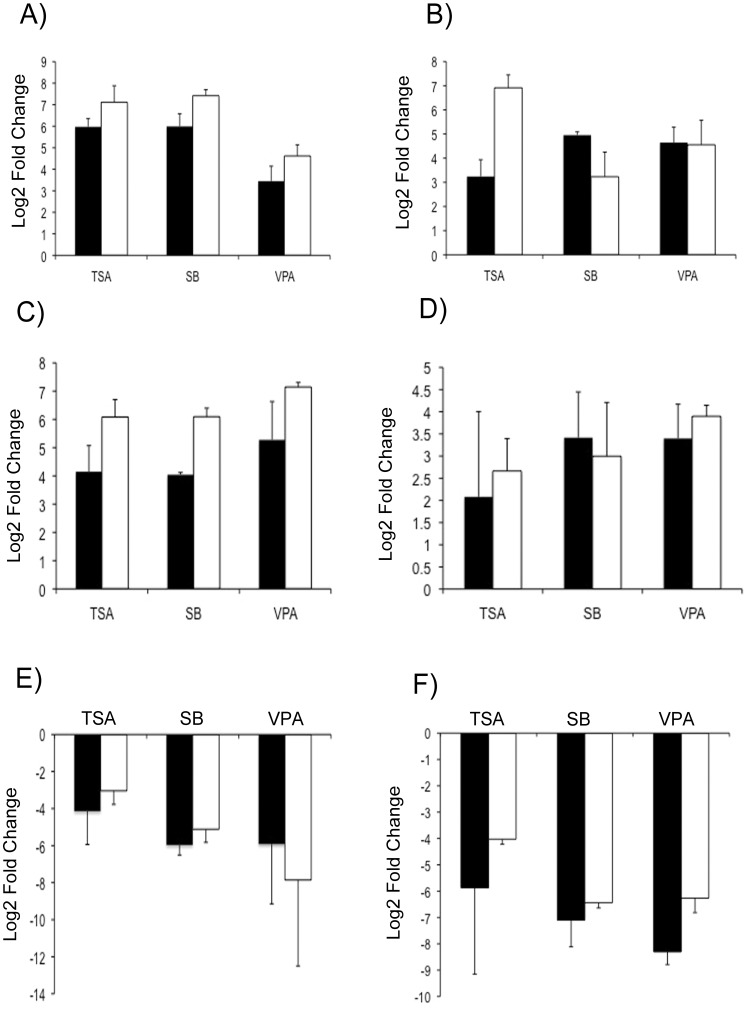
RT-qPCR validation of selected DEGs from the microarray data set. Quantitative RT-PCR was performed to validate changes in transcript levels for (A) *Roundabout axon guidance receptor, homolog 3* (*Robo3*), (B) *medium polypeptide of neurofilament* (*Nfm*), (C) *Homeobox protein a10* (*Hoxa10*), (D) orphan nuclear receptor *Nurr1*, (E) *Toll-like receptor 4* (*TLR4*), (F) *glial fibrillary acidic protein* (*GFAP*) as a result of 48 hours of HDACi treatment. Bars represent the mean fold change of three PCR reactions + s.d. (n = 3). The qPCR results were normalized using GAPDH. Black bars represent the fold change in gene expression (treated group compared to control) obtained in the microarray analysis. White bars represent fold changed in gene expression (treated group compared to control) obtained by RT-qPCR.

We next sought to gain an understanding of the major biological functions of the DEGs in the up- and down-regulated groups. In order to carry out this analysis the web database GENECODIS was used to group genes according to gene ontology (GO) terms [18]. Analysis of GO terms associated with biological processes revealed that in the common up-regulated group of DEGs there was a predominance of genes involved in DNA binding and transcription (Figure 3A). DEGs which were down-regulated by treatment with all three drugs, showed a predominance of genes associated with immune function (Figure 3B). To validate the microarray results, a subset of 6 genes that show differential expression as a result of treatment with all three drugs were selected for RT-qPCR analysis. The genes chosen for validation include *Toll-like receptor 4* (*TLR4*), *Roundabout axon guidance receptor, homolog 3* (*Robo3*), *Homeobox protein a10* (*Hoxa10*), *medium polypeptide of neurofilament* (*Nfm*), *glial fibrillary acidic protein* (*GFAP*), and the orphan nuclear receptor *Nurr1*. Changes in gene expression of DEGs were normalized against the levels of glyceraldehyde 3-phosphate dehydrogenase (GAPDH) expression. Figure 4 shows that for all 6 genes, the significant up- or down-regulation that was determined by microarray analysis was also seen when qPCR was performed. However, in most instances, the fold changes obtained by qPCR were higher than the fold changes in expression seen in the microarray analysis. This observation can be attributed to the higher sensitivity of qPCR. Interestingly, genes associated with a neuronal phenotype (*Nfm* and *Nurr1*) showed strong up-regulation of transcript abundance, while the astrocytic marker GFAP showed strong down-regulation of transcript abundance. This contrasts previous immunohistochemical data, which shows that HDACi treatment results in the loss of midbrain neuronal cells, but does not affect the population of GFAP-positive astrocytes [7]. This difference may be related to the differential stability of proteins versus RNA transcript abundance (see below for additional comments).

**Figure 5:**
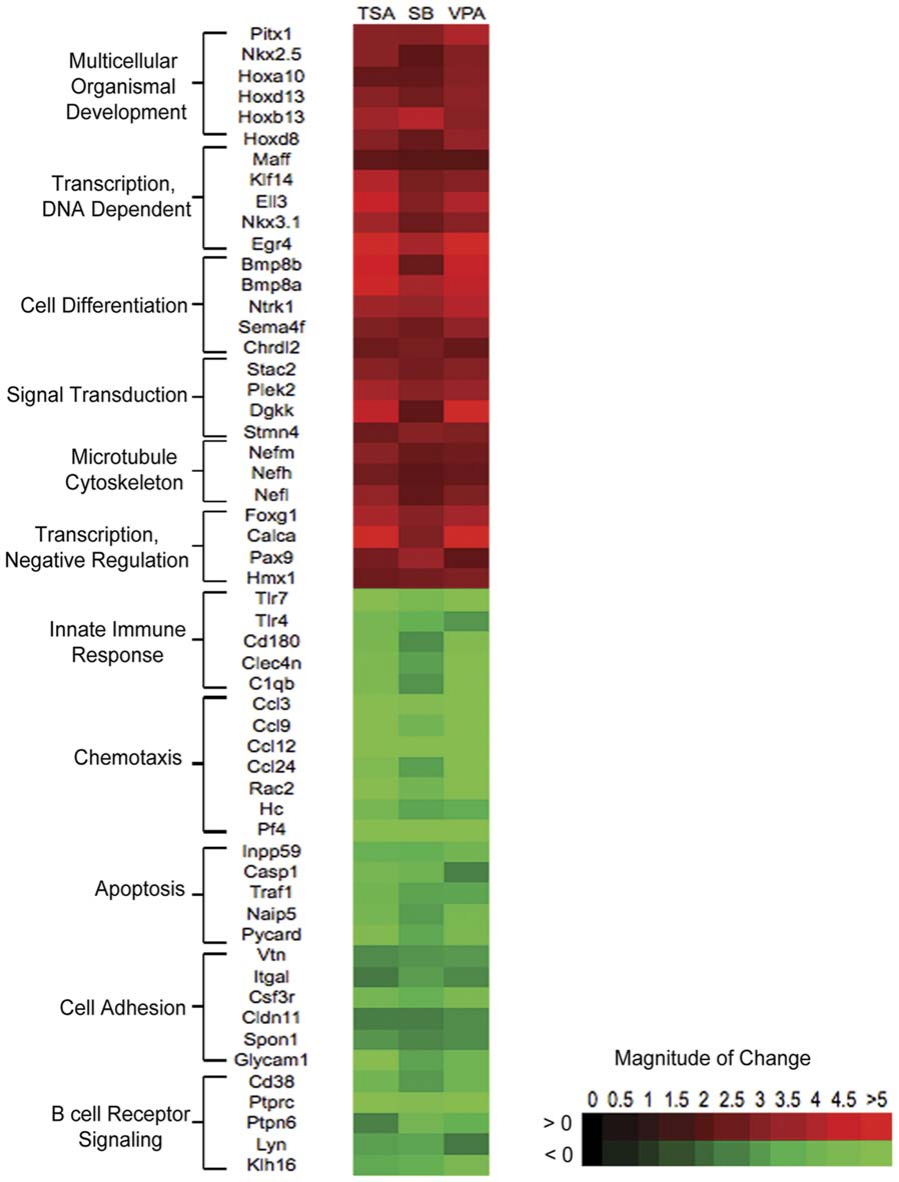
Summary of HDACi-induced expression changes for selected genes associated with GO terms. A representative sampling of GO terms was selected, and a subset of the genes associated with each GO term was plotted on a heat map to show relative expression of the genes of interest. Green =  down-regulated genes, red =  Up-regulated genes.

The heat map in Figure 5 represents some of the genes annotated to selected GO terms. Up-regulated genes are associated with diverse processes such as development and DNA binding. The GO term for multi-cellular organismal development is annotated to a number of Hox genes. HDACi-induced up-regulation of Hox genes in midbrain cells provides an interesting observation given that Hox genes are not expressed in mature midbrain neurons. Other key differentiation genes that are up-regulated by HDACi treatment include BMPs, which is consistent with previous findings [19]. Up-regulated genes are also predominantly associated with the regulation of transcription. Therefore, in all categories, including those not associated with transcription, there are a large number of transcription factors. Interestingly, up-regulated genes are annotated to the GO term for negative regulation of transcription. Therefore, up-regulation of transcription related genes could both induce and repress transcription.

Previous work carried out in relatively pure populations of macrophages and glial cells has shown that HDACi treatment down-regulates the expression of factors associated with innate immunity [20], [21]. Our microarray analysis confirms these observations, and extends them by showing that HDACi treatment down-regulates immune factors in a heterogeneous culture of primary midbrain cells. All three HDACi down-regulate the expression of genes associated with the detection of microbial molecules such as *TLR-4* and *-7*, C-type lectins (*Clec4n*), members of the complement cascade (*C1qb*), and genes involved in B cell receptor signaling. HDACi treatment also down-regulates a number of factors involved in chemotaxis such as chemokines (*Ccl3*, *-9*, -*12*, *-24*), and genes required for cell adhesion (*Vtn, Itgal, Csf3r, Cldn11, Spon11* and *Glycam1*). Chemotaxis and cell adhesion are part of wound healing responses, therefore these categories are also associated with inflammatory and immune responses. Finally, a number of genes annotated to the GO term for apoptosis are down-regulated (*Inpp59, Casp1, Traf1, Naip5*, and *Pycard*). This observation is interesting given the increase in neuronal cell death, but not astrocyte cell death, resulting from HDACi treatment.

### HDAC Inhibitor Treatment Results in a Decrease in the Number of Iba1 Positive Microglia in Midbrain Cultures

**Figure 6:**
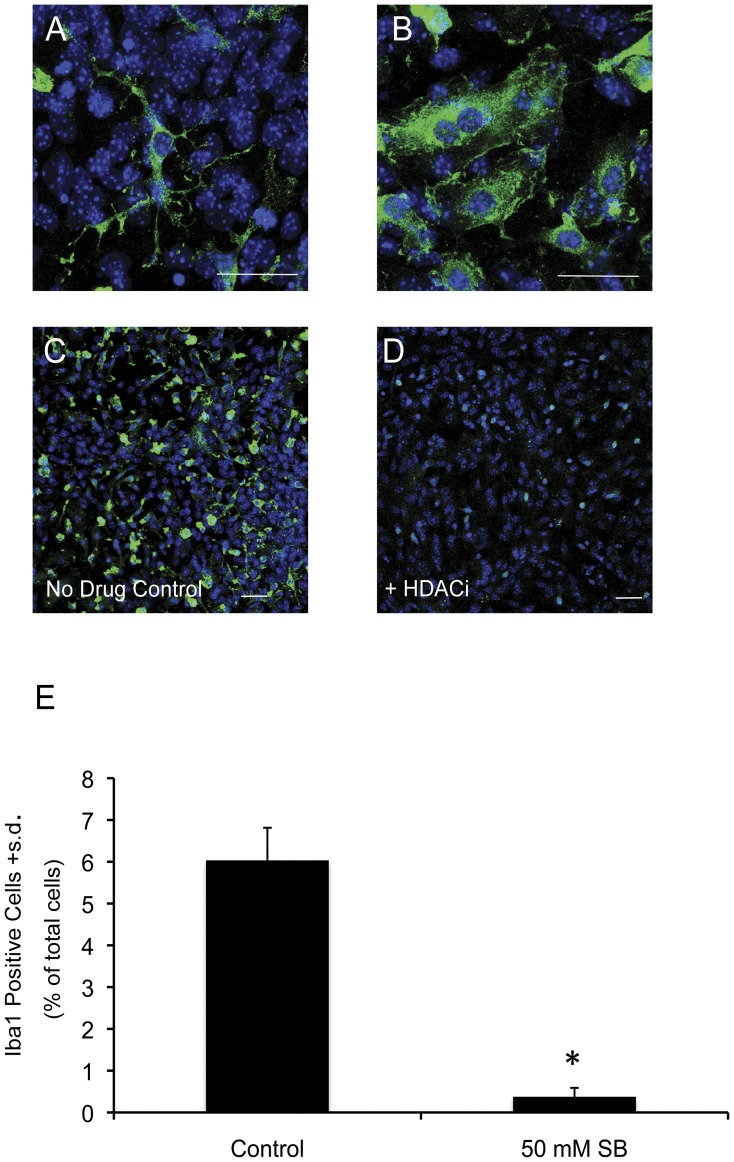
Effects of HDACi treatment on microglia in midbrain cultures. ( A) Iba1 positive microglial cell displaying a ramified morphology. (B) Iba1 positive amoeboid microglia. (C) Iba1 positive cells in an untreated, control midbrain culture. (D) Treatment with 50 mM SB for 48 hours significantly reduces the number of Iba1 positive cells in midbrain cultures. (E) Quantification of the decrease in Iba1 positive cells as a result of HDACi treatment. Bars represent the mean + s.d. (n = 4). Asterisk (*) indicates that SB only treatment group is significantly different (p<0.05) from the control treated group as determined by an unpaired Student’s t-test. Scale bars = 50 µM. Blue =  DAPI positive nuclei, green = Iba1.

Based on the microarray data that indicated a strong down-regulation of innate immune-related factors we next analyzed the effect of HDACi treatment on the main mediator of innate immunity in the brain – microglia. This analysis was first aimed at characterizing the microglial population in differentiated midbrain cultures. We analyzed the microglial specific marker ionized calcium binding adapter molecule (Iba1), which is an EF hand containing protein that is up-regulated upon microglial activation. Under our culture conditions, Iba1 positive cells make up anywhere from 6–14% of the total population of cells in control cultures. The morphology of Iba1+ cells in control cultures is variable, but consistent with the various forms of microglial morphology, with some amoeboid-like cells and other cells appearing to have more ramified processes (Figure 6A–B). Iba1 expression in control- and HDACi-treated midbrain cultures revealed that treatment with SB for 48 hours almost completely eliminated the population of microglial cells in these cultures (Figure 6C–E). HDACi treatment does not appear to drastically change the morphology of the remaining microglia, although this is difficult to assess given that so few microglia remain. Thus, in addition to neurons, midbrain microglial cells appear to be dependent on HDAC activity for their survival *in vitro.*

**Figure 7:**
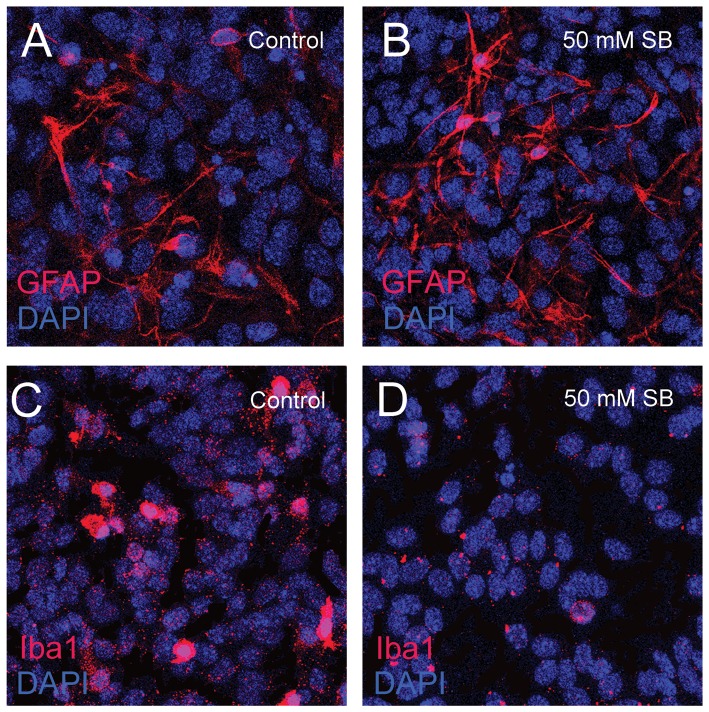
Identification of GFAP-expressing astrocytes and Iba1-expressing microglia after 24 hours of HDACi treatment. Similar numbers of GFAP positive astrocytes (red) in a control midbrain culture (A) compared to cultures treated with 50 mM SB for 24 hours (B). The same treatment reduces the number of Iba1 positive cells in midbrain cultures compared to controls (C, D). Blue = DAPI positive nuclei.

The loss of Iba1+ microglia and decrease in the expression of innate immune related genes (e.g. *TLR4*) correlates with the loss of neurons at 48 hours post-HDACi treatment. To test whether these changes in gene expression and microglia prefigure the loss of neurons, we analyzed the number of Iba1+ microglia at 24 hours after 50 mM SB treatment and also assessed the level of TLR4 expression in the cultures at the same time using RT-qPCR. We previously reported that the numbers of neurons and astrocytes are unchanged after only 24 hours of HDACi treatment [7]. Here, we confirmed that there is no change in the number of GFAP+ astrocytes after 24 hours of SB treatment (Figure 7A, B); however, there was an apparent decrease of Iba1+ microglia in the same cultures (Figure 7C, D), although this decrease was less than what is observed after 48 hours of treatment. We also found that after 24 hours of SB treatment, the level of *TLR4* expression was reduced by almost 4 fold (−3.6±0.9; n = 3) compared to the control treated cultures. Thus, it appears that a decrease in the numbers of microglia and TLR expression precedes the loss of neurons in HDACi treated midbrain cultures.

### The Role of Toll-like Signaling in Modulating the Effects of HDAC Inhibition on Midbrain Neurons

**Figure 8:**
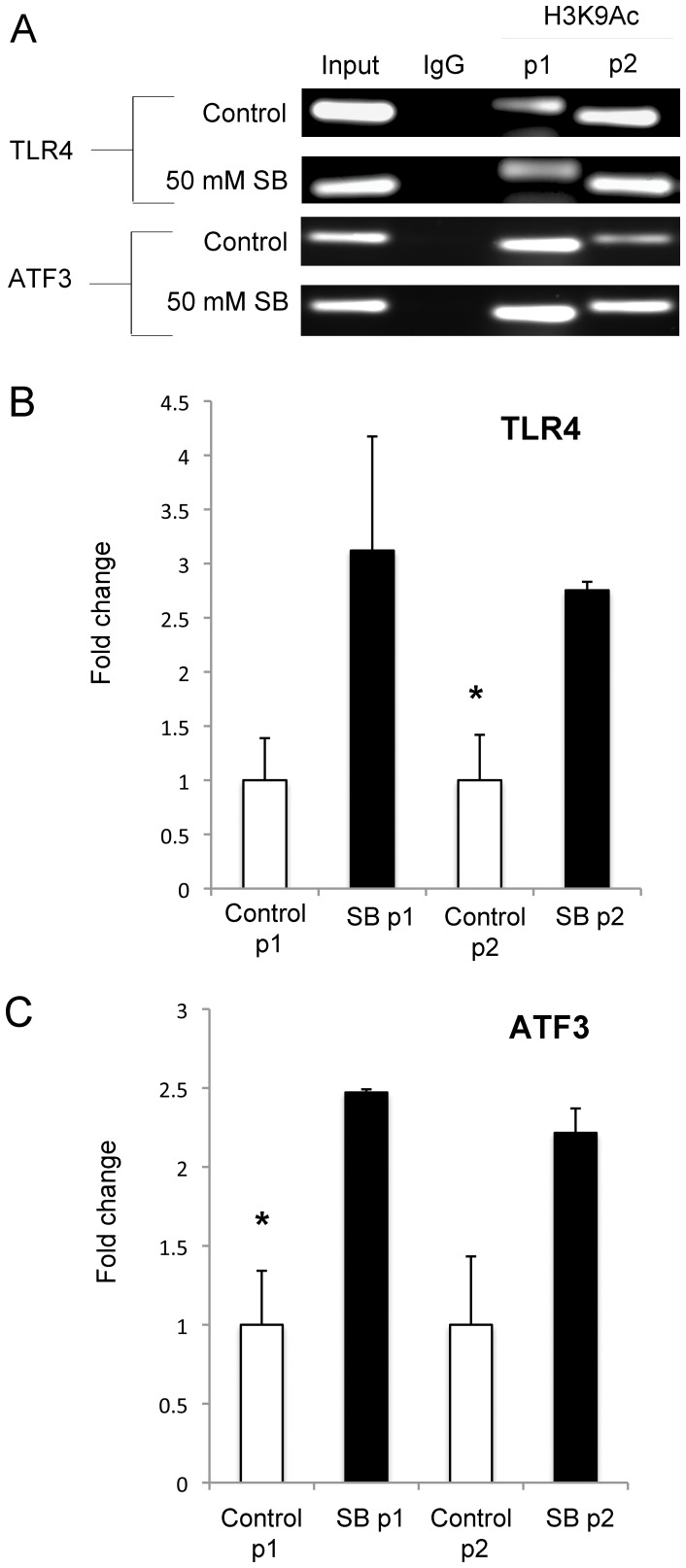
ChIP-qPCR at the *TLR4* and *ATF3* loci. (A) Representative results of qPCR experiments amplifying two separate regions of the *TLR4* and *ATF3* gene promoters (separate p1 and p2 primer sets for each gene) after enrichment of H3K9Ac-DNA complexes from cells obtained after SB treatment or controls. Input =  purified DNA before selection; IgG =  control for non-specific antibody binding to DNA. Quantitative PCR was used to determine that the TLR4 locus (B) and the ATF3 locus (C) were enriched for H3K9Ac at two separate promoter locations after 48 hours of SB treatment. Bars represent the mean fold change + s.d. (n = 3). Asterisk (*) indicates that SB treatment group is significantly different (p<0.05) from the control treated group as determined by an unpaired Student’s t-test.

We previously demonstrated that HDACi treatment dose-dependently enhanced histone H3 (Lys 9) acetylation (H3K9Ac) levels in midbrain cultures [7]. This change is often correlated with a permissive chromatin environment to promote gene transcription. However, the decrease in the transcript levels for immune and other associated genes broadly suggests two possible mechanisms of HDACi action. First, although HDACi broadly enhances H3K9Ac levels in the genome of midbrain cells, it might be possible that a subset of gene loci may instead have a decrease in H3K9Ac levels, which would correlate with the decrease in mRNA levels. Second, HDACi treatment may affect transcript abundance primarily through changes in mRNA stability, instead of changes at the level of transcription. To help distinguish between these two possibilities, we used ChIP-qPCR for two genes, *TLR4* and *ATF3* that were identified as down-regulated and up-regulated, respectively, in our microarray analysis. We immunoprecipitated DNA-protein complexes at these two gene loci using anti-H3K9Ac antibodies from midbrain cultures treated with 50 mM SB for 48 hours or control cultures, and then used two primer sets to PCR amplify regions of the *TLR4* or *ATF3* gene promoters. As shown in Figure 8A, both primer sets (p1 and p2) for the *TLR4* and *ATF3* genes could specifically amplify DNA precipitated with the anti-H3K9Ac antibody in either control or SB treated cultures. Our data indicate that in the midbrain cell population, H3K9Ac levels were enhanced within the gene promoters after 48 hours of SB treatment (Figure 8B, C). Thus, HDACi treatment is indeed correlated with histone hyperacetylation at both the *TLR4* and *ATF3* loci, but this chromatin mark does not necessarily correlate with enhanced mRNA levels. These findings suggest that decreased mRNA stability might be a possible mechanism to account for the changes in *TLR4* transcript abundance, but further experiments are required to support this model.

**Figure 9:**
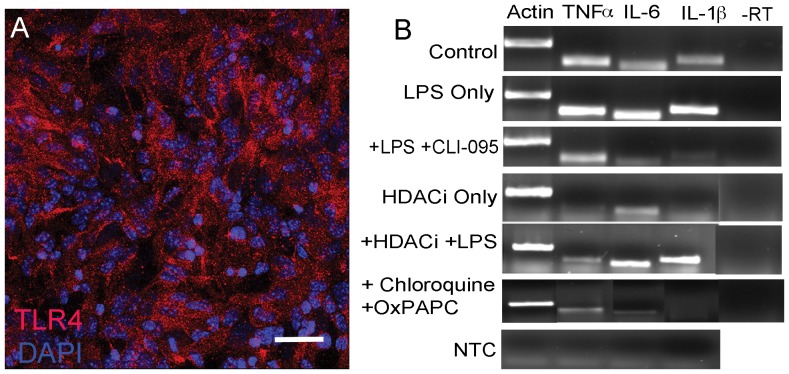
Components of Toll-like signaling in midbrain cultures. (A) TLR4 (red) expression in differentiated midbrain cultures. Nuclei are labeled with DAPI (blue). (B) The effects of HDACi (50 mM SB) and Toll-like signaling agonists and antagonists on the expression of down-stream products of Toll-like signaling (TNFα, IL-6, and IL-1β) in midbrain cultures. Control indicates midbrain cultures that were not treated with any drug. Treatment with the TLR4 agonist LPS increases the levels of all down-stream products of Toll-like signaling. The effects of LPS on Toll-like signaling can be blocked with the TLR4 antagonist CLI-095. HDACi treatment decreases the expression of TNFα and IL-1β. Co-treatment of midbrain cultures with HDACi and LPS rescues the effects of HDACi on IL-1β expression. Treatment with the Toll-like signaling antagonists Chloroquine and OxPAPC results in down regulation of TNFα, IL-6 and IL-1β. No template control (NTC) was performed to rule out contamination of the PCR reactions. All RNA samples were used in a cDNA amplification reaction that did not contain reverse transcriptase (-RT), to rule out contamination with genomic DNA. –RT controls were amplified by PCR using β-actin primers. Gel is representative of at least 2 separate experiments. Scale bar = 50 µM.

**Figure 10:**
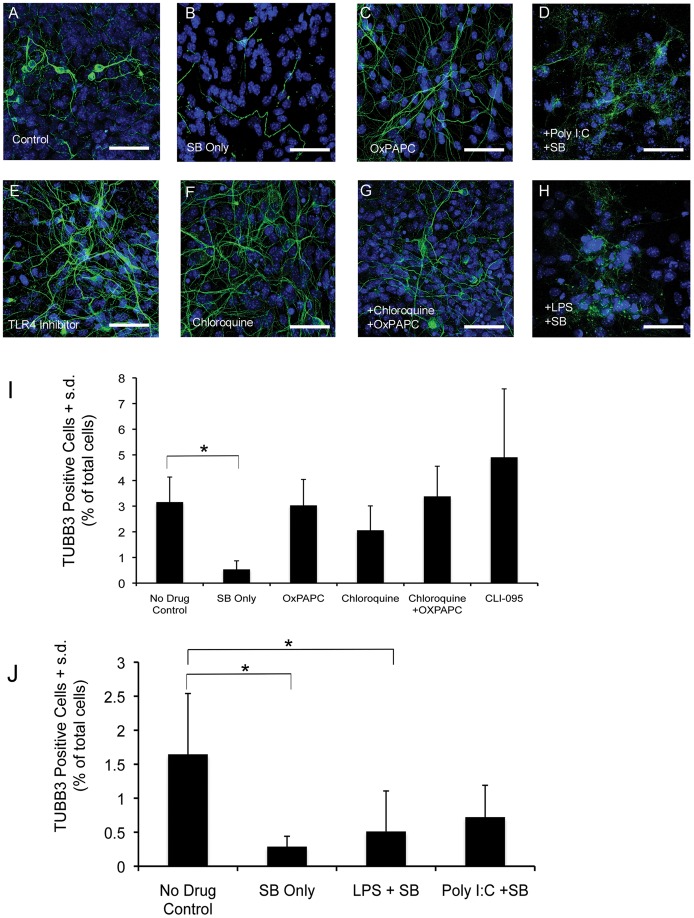
Effects of manipulating TLR signaling on neuronal survival. (A) No drug treatment control stained with TUBB3 (green) and DAPI (blue). (B) Midbrain cells treated with 50 mM SB for 48 hours. TLR inhibition does not affect TUBB3 positive cells. (C) Control midbrain culture treated with OxPAPC alone for 48 hours. (E) Control cultures treated with a specific inhibitor for TLR4. (F) Control cultures treated with chloroquine an inhibitor of intracellular TLRs. (G) Control cultures treated with a combination of Chloroquine and OxPAPC. TLR agonists do not rescue the effects of HDACi on TUBB3 positive neurons. (D) Midbrain cultures co-treated with the TLR3 agonist Poly I:C and 50 mM SB for 48 hours. (H) Midbrain cultures co-treated with the TLR4 agonist LPS and 50 mM SB for 48 hours. (I) Treatment of control midbrain cultures with different Toll-like signaling antagonists does not cause a significant decrease in the number of TUBB3 positive cells. For all treatments bars represent the mean + s.d. (n = 4). (J) Co-treatment of midbrain cultures with HDACi and Toll-like signaling agonists. Bars represent the mean + s.d. (n = 5) for all groups. Asterisk (*) indicates treatment groups showing significant differences using a one-way ANOVA and a *post hoc* Tukey-Kramer MSD test. Scale bars = 50 µM.

Toll-like signaling is one of the main signaling cascades activated as part of the innate immune response. Microarray analysis demonstrated that HDACi down-regulate the expression of multiple TLRs as well as down-stream components of this pathway. These microarray observations were first confirmed by qPCR, which showed that HDACi treatment decreases the expression of TLR4 (Figure 4). Using immunocytochemistry we show that TLR4 is widely expressed in midbrain cultures (Figure 9A), likely indicating that both neurons and non-neuronal cells express TLR4. Furthermore, semi-quantitative RT-PCR showed that HDACi treatment decreases the expression of the cytokines interleukin-6 (IL-6), interleukin 1β (IL-1β), and tumor necrosis factor a (TNFα), which are down-stream products of active Toll-like signaling (Figure 9B). Therefore, we hypothesized that blocking TLR signaling in control cultures could mimic the effects of HDACi treatment on neurons, and conversely, that stimulating TLR signaling could block the effect of HDAC inhibition on neurons. We attempted to block Toll-like signaling using three different inhibitors including the TLR4 specific inhibitor CLI-095, OxPAPC (TLR2 and TLR4 inhibitor), and Chloroquine (intracellular TLR inhibitor). RT-PCR demonstrated that the treatment of midbrain cultures with TLR4 inhibitor or a combination of OxPAPC and Chloroquine results in decreased expression of IL-6, IL-1β, and TNFα (Figure 9B). Despite the effect of these inhibitors on cytokine gene expression, treatment of control cultures with all three of these compounds individually or in combination was not sufficient to phenocopy the effects of HDACi on neurons (Figure 10 C, E, F, G and I).

Lipopolysaccharide (LPS) is an endotoxin, which binds to the TLR4 receptor to activate Toll-like signaling. LPS treatment of midbrain cultures enhances TLR dependent cytokine gene expression, whereas co-treatment with HDACi and LPS blocks the effect of HDAC inhibition on cytokine gene expression (Figure 9B). Based on this we then tested whether LPS and the TLR3 agonist Polyinosinic-polycytidylic acid (Poly I:C) were able to rescue the effects of SB on neurons. A one-way ANOVA revealed an overall significant effect of treatment group (F = 5.07, p = 0.012). Using a *post hoc* Tukey-Kramer MSD test, only the Poly I:C + SB treatment group was not significantly different from the no drug control group (Figure 9B). These data suggest that single TLR agonists may only have a small effect at protecting neurons against HDACi-induced cell death. Furthermore, multiple TLR receptors may need to be activated simultaneously, or more likely that multiple signaling pathways must converge to impart a robust survival effect.

### The Effect of HDAC inhibition on Midbrain Neurons can be Partially Rescued by Treatment with IL-6

Activation of Toll-like signaling in microglia and astrocytes results in the production of cytokines such as IL-6 and TNF-α [22]. While Toll-like signaling-induced cytokine production is most commonly associated with an innate immune response, there is evidence that IL-6 and TNF-α play an important role in the survival and function of mature neurons [23], [24]. Interestingly, TNF-α and IL-6 are expressed under baseline conditions in midbrain cultures, and HDACi treatment decreases the expression of these factors, as demonstrated by RT-PCR (Figure 9B). As demonstrated above, selective TLR agonist treatment in the presence of HDACi provided at best only a small protective effect on the number of neurons (Figure 10), which could indicate that multiple TLR (and other) signaling pathways might converge on downstream cytokine or chemokine expression to promote neuronal survival. Thus, we asked whether the effects of HDACi on neurons might be more substantially blocked by the addition of recombinant cytokines or chemokines.

**Figure 11:**
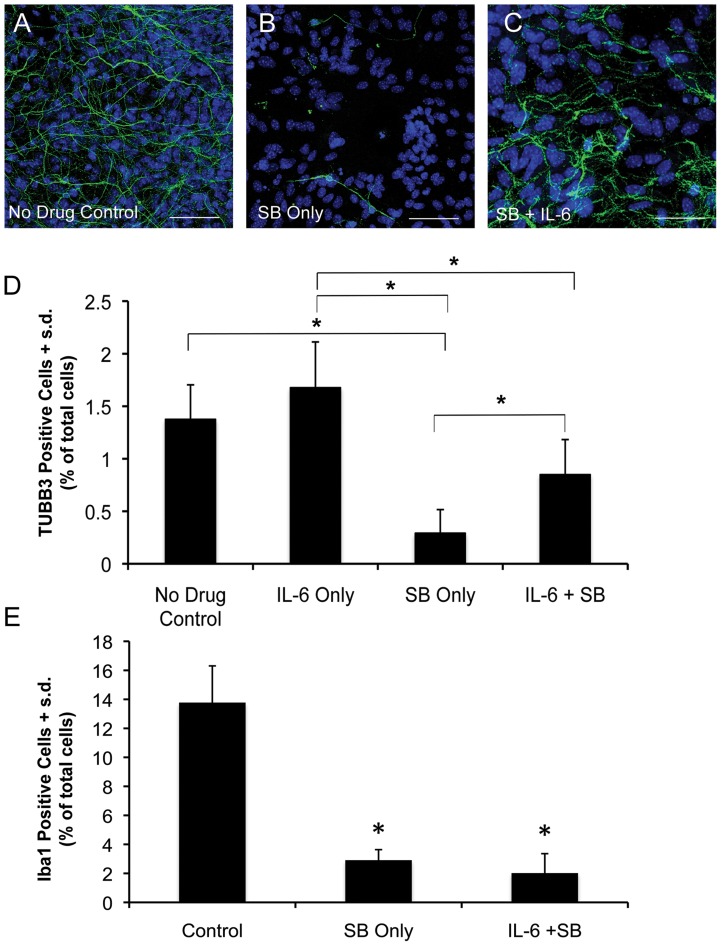
Interleukin-6 (IL-6) treatment partially rescues the effect of HDACi treatment on midbrain neurons. (A) Neurons in an untreated control midbrain culture. (B) Numbers and morphology of midbrain neurons are attenuated following 48 hours of treatment with 50 mM SB. (C) Co-treatment of midbrain cultures with 50 mM SB and IL-6 for 48 hours results in a rescue of the effects of HDACi on neurons. (D) Quantification of the ability of IL-6 to rescue the effects of SB treatment on TUBB3 positive midbrain neurons. Bars represent the mean + s.d. (n = 6) for all groups. Asterisk (*) indicates treatment groups showing significant differences using a one-way ANOVA and a *post hoc* Tukey-Kramer MSD test. (E) IL-6 treatment does not rescue the effects of HDACi on Iba1 positive microglia. Asterisk (*) indicates treatment groups that are significantly different (p<0.05) from no drug control group as determined by an unpaired Student’s t-test. Bar graph represents the mean + s.d. (n = 4). TUBB3 (green), DAPI (blue). Scale bars = 50 µM.

Similar to cytokines, chemokines have been implicated in regulating neuronal survival and function [25]. Microarray analysis revealed that a number of chemokines are strongly down-regulated by treatment with all three drugs (Figure 3B and Table S2). Among this group Ccl3 is one of the most strongly down-regulated chemokines. Based on this evidence, we tested the ability of Ccl3 to rescue the effects of HDAC inhibition on midbrain neurons. Co-treatment with SB and Ccl3 did not produce a rescue effect (data not shown). We also tested the ability of the cytokine IL-6 to rescue the effects of HDACi treatment on neurons (Figure 11A–D). A one-way ANOVA revealed an overall significant effect of treatment group (F = 19.47, p = 3.78×10^−6^). Using a *post hoc* Tukey-Kramer MSD test, we observed that the addition of IL-6 alone to midbrain cultures for 48 hours does not cause a significant increase in the numbers of neurons compared to untreated controls. In contrast, co-treatment of midbrain cultures with SB and IL-6 results in a significant increase in the numbers of neurons compared to SB only treatment. The numbers of neurons in the IL-6+SB group is not significantly lower compared to the untreated control, but it is lower that the IL-6 only group. Thus, IL-6 is alone sufficient to substantially protect against the effects of HDACi treatment on midbrain neurons, but this protective influence may not be complete. Quantification of the numbers of Iba1 positive cells following treatment with 50 mM SB in combination with IL-6 revealed that the number of Iba1 positive cells is significantly decreased despite the presence of IL-6 (Figure 11E), suggesting that the effects of IL-6 on neurons is independent of the presence of microglia.

## Discussion

The aim of this work was to identify factors that are modulated by HDACs and have an effect on the survival of midbrain neurons. Three main conclusions can be drawn from our study. First, HDACi treatment down-regulates the expression of genes associated with innate immunity. Second, Toll-like signaling is active in midbrain cells and selective activation of a least some TLRs has a modest effect on neuronal survival, suggesting that Toll-like signaling works in concert with other pathways. Third, the deleterious effects of HDACi treatment on neuronal survival can be substantially blocked by co-treatment with the cytokine IL-6, which is a downstream target of Toll-like signaling.

The global gene expression profiling analysis was carried out after 48 hours of HDACi treatment to ensure that most neurons and proliferating progenitors were substantially reduced. Under control conditions, neurons make up<5% of the total population of midbrain cells in our cultures, while astrocytes make up ∼40% [7]. Given our findings that microglia (∼10% of cells under control conditions) were also significantly reduced in these cultures, we conclude that the microarray analysis must primarily reflect the status of gene expression in astrocytes after HDACi treatment. Our microarray analysis showed both up-and down-regulation of gene expression, and that 6.8% of the total gene set analyzed was differentially expressed as a result of HDACi treatment. These results correspond with other microarray studies carried out in a variety of different cell types, including relatively pure populations of glial cells, showing the HDACi treatment modulates relatively small subsets of genes and results in both up- and down-regulation of gene expression. Although HDACi treatment is known to cause histone hyperacetylation, our study shows that this occurrence does not necessarily correlate with enhanced transcript levels, as might be expected if the hyperacetylation facilitates transcription. Alternative mechanisms, such as HDAC dependent RNA stability, may account for some of the data we obtained. Regardless, future studies are required to tease-out which of these molecular mechanisms mediates TLR/IL-6 dependent neuronal survival.

**Figure 12:**
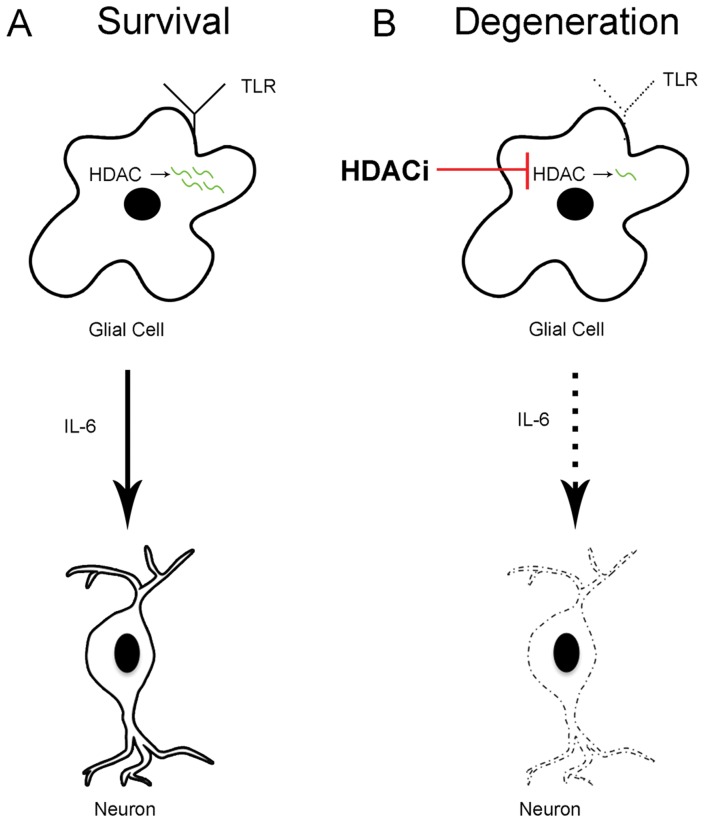
A model of HDAC and TLR signaling in midbrain neuronal survival. A model depicting one possible role of HDACs in mediating neuronal survival, in this case cell non-autonomously. (A) HDAC function in glial cells promotes the stability of mRNA or facilitates translation during the expression of TLR4 and other innate immune related factors. This allows glial cells to respond to TLR signaling and release downstream effectors, such as IL-6 and other factors. These secreted cytokines mediate neuronal survival. (B) HDAC inhibition would lead to hyperacetylation of histones and other proteins, the latter of which might negatively impact mRNA stability or translation of innate immune related genes, such as TLR4. The result is a decrease in overall TLR signaling and release of IL-6 and other cytokines leading to neuronal cell death.

Despite our microarray findings, which are supported by other studies, we cannot rule out the possibility that changes in gene expression, specifically down-regulation, are partly due to the loss of other cell types from our cultures, rather than down-regulation of gene expression in the remaining cells. We examined the expression of *TLR4* after 24 hours of HDACi treatment. At this time point the loss of neurons is not yet evident [7] and we showed here that the loss of microglial cells is noticeable, but is not maximized until 48 hours of HDACi treatment. RT-qPCR analysis demonstrated that even in the presence of neurons, some microglia and normal numbers of astrocytes the levels of *TLR4* are decreased at 24 hours of HDACi treatment. This data supports our assertion that HDACi treatment affects neuronal survival via changes in gene expression, but we cannot yet pinpoint exactly which cells are affected. Our immunolabeling data show that TLR4 is broadly expressed in the midbrain cells, mostly made up of glial cells. Thus, one model is that TLR signaling is required in glial cells (e.g. astrocytes) where HDAC proteins function to promote the stability of TLR and other innate immune related transcripts, ultimately leading to active TLR signaling and the release of IL-6 to promote neuronal survival (Figure 12). Additional experiments will be necessary to clarify exactly which cells receive the TLR signal and which cells produce cytokines that promote neuronal survival.

Microarray analysis also revealed that HDACi treatment results in the up- regulation of numerous transcription factor genes in astrocytes, such as Hox proteins, estrogen receptors, LIM homeodomain, Kruppel-like, and Forkhead-box transcription factors (Table S1). These factors are all involved in general biological processes that are fundamental to cellular development and homeostasis. This observation suggests the interesting possibility that HDACi treatment indirectly causes changes in gene expression via its effects on transcription factor expression, and that this could in turn affect neuronal survival. In order to determine the effects of the up-regulation of these transcription factors by HDAC inhibition, further work should be aimed at identifying their down-stream targets.

We propose that HDACi-induced down-regulation of the Toll-like signaling pathway [26], and other cytokines may influence neuronal survival. LPS treatment directly stimulates Toll-like signaling via an interaction with TLR4 and recent evidence for the influence of HDAC activity on Toll-like signaling has been provided by studies that examine the effects of HDACi treatment on the expression of LPS induced signaling. For example, HDACi treatment of human astrocytes and microglia stimulated with LPS reduces the expression of TLR-induced cytokines, chemokines, and anti-viral genes [27]. Furthermore, the HDAC inhibitors VPA and SB can block LPS signaling in midbrain cultures [28]. One difference from this latter study and our results is the finding that HDAC inhibitors protected dopamine neurons from the neurotoxic effects of pro-inflammatory cytokines, whereas we showed that HDAC inhibitors caused neuronal cell death [7]. The reason for this discrepancy is not clear, but there are significant differences in the cell culture paradigm that was used between the studies, which probably contribute to the duality of neuroimmune effects in the nervous system that are known to be neuroprotective or neurotoxic depending on the cellular context [29]. Nonetheless, our present study provides novel evidence that HDACs are involved in regulating the expression of immune factors in cells derived from the mouse midbrain. In addition to the role of Toll-like signaling in modulating the immune response, this pathway contributes to neuronal function and homeostasis by regulating processes such as neuron development and viability [22], [29]. Other studies have shown that treatment with HDACi can impair the function of the neuroimmune system by down-regulating components of the Toll-like signaling pathway, thus interfering with the innate immune response to invading pathogens [21]. Based on this evidence that HDACi treatment can negatively affect the primary function of the neuroimmune system, via an effect on Toll-like signaling, we posit that HDAC inhibition can also interfere with the role of Toll-like signaling in neuronal survival. Neurons, astrocytes and microglia express Toll-like receptors, however microglia and to some extent astrocytes have been characterized as the main mediators of innate immunity in the brain by promoting neuronal survival through the release of trophic factors and cytokines such as IL-6 [30], [31].

Neuronal survival, development, and function are strongly influenced by IL-6, a member of the neuropoietic cytokine family, which was originally identified in the immune system, and is produced by neurons, astrocytes and microglia in the CNS [24]. One of the main products of activated Toll-like signaling is inflammatory cytokines, including IL-6 [32]. Therefore, we attempted to block the effects of HDACi treatment on neurons by separately up-regulating Toll-like signaling with the TLR agonists LPS and Poly I:C. Previous studies have established that LPS and Poly I:C treatment of primary glial cultures results in stimulation of Toll-like signaling [33], [34]. In our cultures, RT-PCR demonstrated that LPS up-regulated the expression of IL-6, IL1β, and TNF-α, down-stream products of Toll-like signaling. Treatment with selective TLR agonists does seem to cause a slight increase in the numbers of neurons in HDACi-treated cultures. Therefore, the manipulation of Toll-like signaling at the level of single receptors is likely not sufficient to robustly promote neuronal survival. In contrast, the application of recombinant IL-6 to HDACi-treated cultures was able to substantially rescue the effects of HDACi treatment on midbrain neurons. This result suggests that although IL-6 confers a neuroprotective effect, activation of more than one TLR is necessary to generate the levels of IL-6 required for this effect, at least during the time interval used for testing in our assays (48 hours). It is possible that extended direct TLR signaling is required to achieve neuroprotection. Alternatively, different pathways in addition to TLR signaling might be involved in the production of IL-6. The production of IL-6 can be stimulated by pro-inflammatory cytokines such as TNF-α, viral and bacterial pathogens, and neurotransmitters such as norepinephrine [35]. The stimulation of IL-6 synthesis by neurotransmitters suggests that cross talk between neurons and glia is essential for the maintenance of proper IL-6 levels. One possibility is that the loss of neurons in HDACi-treated cultures likely reduces the levels of IL-6 by interfering with neuron-glia interactions. In conclusion, this work provides further evidence that the neuroimmune system is involved in the maintenance of midbrain neurons, and that HDACs play a role in the proper expression and functioning of neuroimmune factors to promote neuronal survival.

## Supporting Information

Table S1 **Common up-regulated genes in response to either TSA, SB, or VPA.**
(DOCX)

Table S2 **Common down-regulated genes in response to either TSA, SB, or VPA.**
(DOCX)

## References

[1] Sofroniew MV, Vinters HV (2010). Astrocytes: Biology and pathology.. Acta Neuropathol.

[2] Shih AY, Johnson DA, Wong G, Kraft AD, Jiang L (2003). Coordinate regulation of glutathione biosynthesis and release by Nrf2-expressing glia potently protects neurons from oxidative stress.. J Neurosci.

[3] Barreto GE, Gonzalez J, Torres Y, Morales L (2011). Astrocytic-neuronal crosstalk: Implications for neuroprotection from brain injury.. Neurosci Res.

[4] Allaman I, Belanger M, Magistretti PJ (2011). Astrocyte-neuron metabolic relationships: For better and for worse.. Trends Neurosci.

[5] Liberto CM, Albrecht PJ, Herx LM, Yong VW, Levison SW (2004). Pro-regenerative properties of cytokine-activated astrocytes.. J Neurochem.

[6] Farina C, Aloisi F, Meinl E (2007). Astrocytes are active players in cerebral innate immunity.. Trends Immunol.

[7] Forgione N, Tropepe V (2011). Histone deacetylase inhibition promotes caspase-independent cell death of ventral midbrain neurons.. Mol Cell Neurosci.

[8] Broide RS, Redwine JM, Aftahi N, Young W, Bloom FE (2007). Distribution of histone deacetylases 1–11 in the rat brain.. J Mol Neurosci.

[9] MacDonald JL, Roskams AJ (2008). Histone deacetylases 1 and 2 are expressed at distinct stages of neuro-glial development.. Dev Dyn.

[10] Montgomery RL, Hsieh J, Barbosa AC, Richardson JA, Olson EN (2009). Histone deacetylases 1 and 2 control the progression of neural precursors to neurons during brain development.. Proc Natl Acad Sci U S A.

[11] Kazantsev AG, Thompson LM (2008). Therapeutic application of histone deacetylase inhibitors for central nervous system disorders.. Nat Rev Drug Discov.

[12] Graeber MB, Streit WJ (2010). Microglia: Biology and pathology.. Acta Neuropathol.

[13] Rivest S (2009). Regulation of innate immune responses in the brain.. Nat Rev Immunol.

[14] Langley B, D'Annibale MA, Suh K, Ayoub I, Tolhurst A (2008). Pulse inhibition of histone deacetylases induces complete resistance to oxidative death in cortical neurons without toxicity and reveals a role for cytoplasmic p21(waf1/cip1) in cell cycle-independent neuroprotection.. J Neurosci.

[15] LaBonte MJ, Wilson PM, Fazzone W, Groshen S, Lenz HJ (2009). DNA microarray profiling of genes differentially regulated by the histone deacetylase inhibitors vorinostat and LBH589 in colon cancer cell lines.. BMC Med Genomics.

[16] Chiba T, Yokosuka O, Fukai K, Kojima H, Tada M (2004). Cell growth inhibition and gene expression induced by the histone deacetylase inhibitor, trichostatin A, on human hepatoma cells.. Oncology.

[17] Fukuchi M, Nii T, Ishimaru N, Minamino A, Hara D (2009). Valproic acid induces up- or down-regulation of gene expression responsible for the neuronal excitation and inhibition in rat cortical neurons through its epigenetic actions.. Neurosci Res.

[18] Carmona-Saez P, Chagoyen M, Tirado F, Carazo JM, Pascual-Montano A (2007). GENECODIS: A web-based tool for finding significant concurrent annotations in gene lists.. Genome Biol.

[19] Shaked M, Weissmuller K, Svoboda H, Hortschansky P, Nishino N (2008). Histone deacetylases control neurogenesis in embryonic brain by inhibition of BMP2/4 signaling.. PLoS One.

[20] Suh HS, Choi S, Khattar P, Choi N, Lee SC (2010). Histone deacetylase inhibitors suppress the expression of inflammatory and innate immune response genes in human microglia and astrocytes.. J Neuroimmune Pharmacol.

[21] Roger T, Lugrin J, Le Roy D, Goy G, Mombelli M (2011). Histone deacetylase inhibitors impair innate immune responses to toll-like receptor agonists and to infection.. Blood.

[22] Okun E, Griffioen KJ, Mattson MP (2011). Toll-like receptor signaling in neural plasticity and disease.. Trends Neurosci.

[23] Park KM, Bowers WJ (2010). Tumor necrosis factor-alpha mediated signaling in neuronal homeostasis and dysfunction.. Cell Signal.

[24] Gadient RA, Otten UH (1997). Interleukin-6 (IL-6)–a molecule with both beneficial and destructive potentials.. Prog Neurobiol.

[25] Semple BD, Kossmann T, Morganti-Kossmann MC (2010). Role of chemokines in CNS health and pathology: A focus on the CCL2/CCR2 and CXCL8/CXCR2 networks.. J Cereb Blood Flow Metab.

[26] Okun E, Griffioen KJ, Mattson MP (2011). Toll-like receptor signaling in neural plasticity and disease.. Trends Neurosci.

[27] Suh HS, Choi S, Khattar P, Choi N, Lee SC (2010). Histone deacetylase inhibitors suppress the expression of inflammatory and innate immune response genes in human microglia and astrocytes.. J Neuroimmune Pharmacol.

[28] Chen PS, Wang CC, Bortner CD, Peng GS, Wu X (2007). Valproic acid and other histone deacetylase inhibitors induce microglial apoptosis and attenuate liopolysaccharide-induced dopaminergic neurotoxicity.. Neuroscience.

[29] Glezer I, Simard AR, Rivest S (2007). Neuroprotective role of the innate immune system by microglia.. Neuroscience.

[30] Hama T, Kushima Y, Miyamoto M, Kubota M, Takei N (1991). Interleukin-6 improves the survival of mesencephalic catecholaminergic and septal cholinergic neurons from postnatal, two-week-old rats in cultures.. Neuroscience.

[31] Kushima Y, Hatanaka H (1992). Interleukin-6 and leukemia inhibitory factor promote the survival of acetylcholinesterase-positive neurons in culture from embryonic rat spinal cord.. Neurosci Lett.

[32] Kawai T, Akira S (2010). The role of pattern-recognition receptors in innate immunity: Update on toll-like receptors.. Nat Immunol.

[33] Faraco G, Pittelli M, Cavone L, Fossati S, Porcu M (2009). Histone deacetylase (HDAC) inhibitors reduce the glial inflammatory response in vitro and in vivo.. Neurobiol Dis.

[34] Steelman AJ, Li J (2011). Poly(I:C) promotes TNFalpha/TNFR1-dependent oligodendrocyte death in mixed glial cultures.. J Neuroinflammation.

[35] Norris JG, Benveniste EN (1993). Interleukin-6 production by astrocytes: Induction by the neurotransmitter norepinephrine.. J Neuroimmunol.

