# Supplementary material for: Toll-Like Signaling and the Cytokine IL-6 Regulate Histone Deacetylase Dependent Neuronal Survival
Source: PLoS One. 2012 Jul 27;7(7):e41033. doi: 10.1371/journal.pone.0041033 (PMC3407143; doi:10.1371/journal.pone.0041033)
Supplement: Table S1 — Common up-regulated genes in response to either TSA, SB, or VPA. (DOCX) [file pone.0041033.s001.docx]

**Supplement 1: Up Regulated Genes**

| UID | Gene | Description | TSA | SB | VPA |
| --- | --- | --- | --- | --- | --- |
| NM_029597 | 1700018B08Rik | n/a | 3.213744 | 2.671303 | 2.238619 |
| AK016569 | 4932442L08Rik | n/a | 2.876759 | 2.675095 | 2.2378 |
| NM_001033304 | 5330417C22Rik | n/a | 3.623316 | 0.898817 | 3.800932 |
| NM_183152 | 6330514A18Rik | n/a | 4.07384 | 2.516239 | 3.762933 |
| AK048964 | A230070E04Rik | n/a | 2.380707 | 2.118365 | 2.338416 |
| AK145690 | Aim1l | n/a | 3.196356 | 3.120676 | 2.849342 |
| AK043041 | AK043041 | n/a | 2.173569 | 2.682568 | 2.438934 |
| AK086786 | AK086786 | n/a | 2.109652 | 2.461926 | 2.88861 |
| NM_007439 | Alk | anaplastic lymphoma kinase | 2.485145 | 2.008088 | 2.5418 |
| NM_011786 | Aloxe3 | arachidonate lipoxygenase 3 | 2.191294 | 1.986686 | 2.014661 |
| NM_175270 | Ankrd56 | ankyrin repeat domain 56 | 4.90874 | 2.736309 | 4.864422 |
| NM_183027 | Ap1s3 | AP-1 complex subunit sigma-3 | 2.925963 | 1.882009 | 3.10788 |
| NM_018790 | Arc | activity regulated cytoskeletal-associated protein | 3.111934 | 4.053845 | 2.775407 |
| NM_001039645 | Asphd1 | aspartate beta-hydroxylase domain containing 1 ( | 2.749028 | 2.088474 | 3.100958 |
| NM_007526 | Barx1 | BarH-like homeobox 1 | 5.914676 | 3.514771 | 6.113404 |
| NM_007558 | Bmp8a | bone morphogenetic protein 8a | 4.828468 | 3.769271 | 4.344735 |
| NM_007559 | Bmp8b | bone morphogenetic protein 8b | 4.648948 | 2.426032 | 4.481241 |
| NM_175235 | Brunol6 | bruno-like 6, RNA binding protein | 3.617766 | 3.71704 | 3.849438 |
| NM_175938 | Btn2a2 | butyrophilin, subfamily 2, member A2 | 2.299652 | 2.575223 | 2.959033 |
| AK082735 | C230098O21Rik | n/a | 2.915164 | 3.165895 | 3.02209 |
| NM_177691 | C79127 | n/a | 4.69349 | 2.411402 | 4.015182 |
| NM_013879 | Cabp1 | calcium binding protein 1 | 2.801963 | 2.185425 | 2.739919 |
| NM_001033954 | Calca | calcitonin/calcitonin-related polypeptide, alpha | 5.181578 | 2.901623 | 5.794004 |
| NM_007587 | Calca | calcitonin/calcitonin-related polypeptide, alpha | 5.181578 | 2.901623 | 5.794004 |
| NM_007662 | Cdh15 | cadherin 15 | 2.861084 | 2.516348 | 2.213103 |
| NM_007673 | Cdx2 | caudal type homeo box 2 | 3.145844 | 2.132122 | 3.52236 |
| NM_007693 | Chga | chromogranin A | 4.576355 | 3.666244 | 4.876693 |
| NM_133709 | Chrdl2 | chordin-like 2 | 2.478005 | 2.729814 | 2.362085 |
| NM_176844 | Chrna5 | cholinergic receptor, nicotinic, alpha polypeptide 5 | 3.835722 | 3.244155 | 4.040573 |
| NM_016685 | Comp | cartilage oligomeric matrix protein | 2.754114 | 2.01517 | 2.67381 |
| NM_170684 | Cpne7 | copine VII | 3.878807 | 2.737753 | 3.563916 |
| AK081332 | Dgkk | n/a | 4.386345 | 2.184027 | 5.279344 |
| NM_007857 | Dhh | desert hedgehog | 4.67938 | 2.698818 | 4.822872 |
| NM_170593 | Disp2 | dispatched homolog 2 | 3.249582 | 2.533555 | 3.384487 |
| NM_010090 | Dusp2 | dual specificity phosphatase 2 | 5.23522 | 3.687281 | 3.286831 |
| NM_007899 | Ecm1 | extracellular matrix protein 1 | 2.41203 | 2.083758 | 2.35529 |
| AK020438 | Efna3 | n/a | 3.443561 | 2.576597 | 3.252868 |
| NM_020596 | Egr4 | early growth response 4 | 6.393743 | 3.762126 | 5.956289 |
| NM_145973 | Ell3 | elongation factor RNA polymerase II-like 3 | 4.551627 | 2.948918 | 3.971415 |
| ENSMUST00000096572 | ENSMUST00000096572 | n/a | 2.503623 | 2.491831 | 2.752745 |
| NM_177671 | Epha10 | Eph receptor A10 | 3.555954 | 2.700783 | 2.153595 |
| NM_011934 | Esrrb | estrogen related receptor, beta | 4.343378 | 2.699301 | 4.760608 |
| NM_007957 | Esx1 | extraembryonic, spermatogenesis, homeobox 1 | 4.736379 | 3.841272 | 5.128661 |
| NM_007959 | Etv2 | ets variant gene 2 | 3.020112 | 2.260403 | 3.327122 |
| NM_172857 | Exdl1 | exonuclease 3'-5' domain-like 1 | 2.026651 | 2.116105 | 2.030309 |
| NM_008029 | Flt4 | FMS-like tyrosine kinase 4 | 2.397975 | 2.142258 | 3.241737 |
| NM_008241 | Foxg1 | forkhead box G1 | 3.845412 | 3.066819 | 3.744462 |
| NM_008072 | Gabrd | gamma-aminobutyric acid (GABA-A) receptor, subunit delta | 2.460517 | 2.177647 | 2.065893 |
| NM_013847 | Gcat | glycine C-acetyltransferase (2-amino-3-ketobutyrate-coenzyme A ligase) | 2.592896 | 2.106305 | 2.431126 |
| AK136383 | Gja3 | gap junction membrane channel protein alpha 3 | 4.377345 | 3.476845 | 4.538633 |
| NM_001033302 | Gm129 | gene model 129 | 2.179166 | 2.419446 | 2.460469 |
| NM_001085513 | Gm693 | gene model 693 | 2.777073 | 2.181498 | 2.880934 |
| NM_021347 | Gsdma1 | gasdermin A1 | 2.394012 | 2.175534 | 2.019543 |
| BC010322 | H2-Ab1 | histocompatibility 2, class II antigen A, beta 1, mRNA | 2.998313 | 1.97051 | 3.574749 |
| NM_207105 | H2-Ab1 | histocompatibility 2, class II antigen A, beta 1, mRNA | 2.998313 | 1.97051 | 3.574749 |
| NM_177900 | Hapln4 | hyaluronan and proteoglycan link protein 4 | 3.163154 | 2.238883 | 3.573143 |
| NM_010445 | Hmx1 | H6 homeo box 1 | 2.477564 | 2.655212 | 2.881789 |
| NM_008263 | Hoxa10 | homeo box A10 | 2.349894 | 2.302016 | 3.043341 |
| NM_008267 | Hoxb13 | homeo box B13 | 3.087035 | 2.592452 | 3.198744 |
| NM_008270 | Hoxb9 | homeo box B9 | 2.477417 | 4.541104 | 2.375564 |
| NM_008272 | Hoxc9 | homeo box C9 | 4.134265 | 3.009733 | 5.165076 |
| NM_008275 | Hoxd13 | homeo box D13 | 3.618709 | 4.129443 | 3.080579 |
| NM_008276 | Hoxd8 | homeo box D8 | 3.024914 | 2.359575 | 3.358301 |
| NM_010479 | Hspa1a | heat shock protein 1A | 2.795518 | 2.825814 | 2.201492 |
| NM_008350 | Il11 | interleukin 11 | 3.194868 | 2.908692 | 2.24662 |
| NM_016851 | Irf6 | interferon regulatory factor 6 | 4.062405 | 3.003395 | 3.701542 |
| NM_027397 | Isl2 | insulin related protein 2 | 3.115656 | 2.628039 | 2.911977 |
| NM_146125 | Itpka | inositol 1,4,5-trisphosphate 3-kinase A | 2.493068 | 2.589669 | 2.037571 |
| AK030435 | Klf14 | n/a | 4.079532 | 2.726396 | 3.035657 |
| NM_008499 | Lhx5 | LIM homeobox protein 5 | 3.889976 | 3.628773 | 4.283255 |
| NM_008500 | Lhx6 | LIM homeobox protein 6 | 3.094435 | 2.398906 | 3.21982 |
| NM_001025565 | Lhx9 | LIM homeobox protein 9 | 3.281494 | 1.976206 | 3.569772 |
| NM_001042577 | Lhx9 | LIM homeobox protein 9 | 3.281494 | 1.976206 | 3.569772 |
| NM_011698 | Lin7b | lin-7 homolog B | 3.156124 | 3.483851 | 2.650206 |
| NM_010755 | Maff | v-maf musculoaponeurotic fibrosarcoma oncogene, F | 2.221043 | 2.035565 | 2.036203 |
| AK037524 | Mctp2 | n/a | 3.500875 | 2.679746 | 2.76403 |
| NM_019944 | Mnx1 | motor neuron and pancreas homeobox 1 | 2.901598 | 3.20647 | 3.089136 |
| NAP109359-1 | NAP109359-1 | n/a | 3.027508 | 1.989057 | 3.134104 |
| NM_010904 | Nefh | neurofilament, heavy polypeptide | 2.59724 | 2.158828 | 2.343148 |
| AK149219 | Nefl | n/a | 3.335048 | 2.224902 | 2.885718 |
| NM_008691 | Nefm | neurofilament, medium polypeptide | 3.129607 | 2.390644 | 2.555206 |
| NM_008700 | Nkx2-5 | NK2 transcription factor related 5 | 3.142687 | 2.110159 | 2.970032 |
| NM_010921 | Nkx3-1 | NK-3 transcription factor, locus 1 | 3.66619 | 2.474874 | 3.097579 |
| NM_019515 | Nmu | neuromedin U | 4.799041 | 4.341374 | 5.044434 |
| NM_016789 | Nptx2 | neuronal pentraxin 2 | 2.601253 | 2.593148 | 2.367856 |
| NM_022029 | Nrgn | neurogranin | 3.256522 | 2.176436 | 3.533784 |
| NM_020610 | Nrip3 | nuclear receptor interacting protein 3 | 4.149442 | 3.411358 | 3.986131 |
| NM_001033124 | Ntrk1 | neurotrophic tyrosine kinase, receptor, type 1 | 3.607289 | 3.357372 | 4.067493 |
| NM_194268 | Onecut2 | one cut domain 2 | 2.255712 | 2.741141 | 2.667593 |
| NM_152818 | Osbp2 | oxysterol binding protein 2 | 2.42575 | 2.705382 | 2.29634 |
| NM_008780 | Pax1 | paired box gene 1 | 5.142032 | 4.239118 | 5.016902 |
| NM_011041 | Pax9 | paired box gene 9 | 2.704922 | 3.501745 | 2.227805 |
| NM_198191 | Pip5kl1 | phosphatidylinositol-4-phosphate 5-kinase-like 1 | 3.693354 | 2.763938 | 3.450368 |
| NM_011097 | Pitx1 | paired-like homeodomain transcription factor 1 | 3.122734 | 3.033715 | 3.926973 |
| NM_019762 | Pkp3 | plakophilin 3 | 3.870876 | 2.570974 | 3.768737 |
| NM_013738 | Plek2 | pleckstrin 2 | 3.742351 | 3.074379 | 3.444328 |
| NM_027982 | Ppm1j | protein phosphatase 1J | 3.460729 | 2.288255 | 3.624343 |
| NM_019429 | Prss16 | protease, serine, 16 | 2.618523 | 2.076077 | 2.560169 |
| NM_008985 | Ptprn | protein tyrosine phosphatase, receptor type, N | 3.471258 | 3.183545 | 3.704057 |
| NM_023852 | Rab3c | RAB3C, member RAS oncogene family | 3.266223 | 2.741333 | 3.370628 |
| NM_194055 | Rbm35a | RNA binding motif protein 35A | 2.422872 | 2.169714 | 3.196354 |
| NM_019799 | Rhcg | Rhesus blood group-associated C glycoprotein | 3.042269 | 2.701518 | 3.382701 |
| AK007019 | Rundc3a | n/a | 2.638564 | 2.029237 | 2.11545 |
| NM_011350 | Sema4f | sema domain, immunoglobulin domain (Ig), TM domain, and short cytoplasmic domain | 2.864034 | 2.568434 | 3.293273 |
| NM_008871 | Serpine1 | serine (or cysteine) peptidase inhibitor, E1 | 3.421372 | 3.325775 | 2.731137 |
| NM_146257 | Slc29a4 | solute carrier, 29a4 | 3.142551 | 2.386902 | 3.337175 |
| NM_053248 | Slc5a5 | solute carrier, 5a5 | 3.318324 | 2.296027 | 3.236682 |
| M22012 | Snap25 | synaptosomal-associated protein 25 | 4.36622 | 2.383414 | 4.380449 |
| NM_011428 | Snap25 | synaptosomal-associated protein 25 | 4.36622 | 2.383414 | 4.380449 |
| NM_016907 | Spint1 | serine protease inhibitor | 2.942012 | 2.136382 | 3.174686 |
| NM_146028 | Stac2 | SH3 and cysteine rich domain 2 | 3.066405 | 2.632735 | 3.020182 |
| NM_019675 | Stmn4 | stathmin-like 4 | 2.517945 | 3.048906 | 2.893501 |
| NM_172440 | Stxbp5l | syntaxin binding protein 5-like | 3.798543 | 2.080041 | 2.915277 |
| NM_013873 | Sult4a1 | sulfotransferase family 4A, 1 | 3.63888 | 3.119514 | 3.981433 |
| NM_008532 | Tacstd1 | tumor-associated calcium signal transducer 1 | 5.19041 | 3.985756 | 4.714645 |
| TC1634227 | TC1634227 | n/a | 4.051267 | 2.854969 | 2.409677 |
| NM_009328 | Tcf15 | transcription factor 15 | 2.841631 | 2.913499 | 2.366492 |
| NM_009335 | Tcfap2c | transcription factor AP-2, gamma | 3.63743 | 2.532039 | 3.311789 |
| NM_178254 | Tcfl5 | transcription factor-like 5 | 3.805265 | 3.054306 | 3.685745 |
| NM_031374 | Tex15 | testis expressed gene 15 | 3.259103 | 2.069418 | 3.60545 |
| NM_001044384 | Timp1 | tissue inhibitor of metalloproteinase 1 | 3.054076 | 2.082196 | 3.044071 |
| NM_183311 | Tmem145 | transmembrane protein 145 | 3.574457 | 2.685057 | 3.389851 |
| NM_001001885 | Tmem151a | transmembrane protein 151A | 2.522547 | 2.120164 | 2.207431 |
| NM_025452 | Tmem54 | transmembrane protein 54 | 3.597726 | 4.074257 | 3.832441 |
| NM_182991 | Tmem59l | transmembrane protein 59-like | 4.27585 | 2.543735 | 4.363936 |
| NM_175502 | Tmem74 | transmembrane protein 74 | 2.410572 | 4.165811 | 2.488729 |
| NM_011912 | Vax2 | ventral anterior homeobox containing gene | 3.004366 | 2.360094 | 2.884862 |
