# Supplementary material for: Toll-Like Signaling and the Cytokine IL-6 Regulate Histone Deacetylase Dependent Neuronal Survival
Source: PLoS One. 2012 Jul 27;7(7):e41033. doi: 10.1371/journal.pone.0041033 (PMC3407143; doi:10.1371/journal.pone.0041033)
Supplement: Table S2 — Common down-regulated genes in response to either TSA, SB, or VPA. (DOCX) [file pone.0041033.s002.docx]

Supplement 2: Down-Regulated Genes

| Gene Identifier | Gene Name | Description | TSA | SB | VPA |
| --- | --- | --- | --- | --- | --- |
| AK004424 | 1110069O07Rik | n/a | -3.610513 | -2.715383 | -3.9569 |
| AK005560 | 1600029I14Rik | n/a | -2.758912 | -3.489126 | -3.040155 |
| NM_001081275 | 1700009P17Rik | n/a | -3.286794 | -3.573289 | -3.138748 |
| ENSMUST00000079738 | 1700011I03Rik | n/a | -3.82663 | -2.435266 | -4.205984 |
| NM_029306 | 1700012B09Rik | n/a | -3.206666 | -2.868387 | -2.815706 |
| NM_029604 | 1700027A23Rik | n/a | -2.771616 | -2.785236 | -2.458251 |
| AK007434 | 1810011H11Rik | n/a | -6.61262 | -4.642762 | -6.649582 |
| NM_001013022 | 2010001J22Rik | n/a | -4.956064 | -3.546357 | -4.770895 |
| XM_900828 | 2010003K15Rik | n/a | -3.899669 | -3.192446 | -4.335996 |
| BC032884 | 2210020M01Rik | n/a | -5.003338 | -4.327131 | -5.059462 |
| AK011565 | 2610027F03Rik | n/a | -3.369135 | -2.692904 | -3.047528 |
| XM_001002726 | 3100002J23Rik | n/a | -3.367314 | -3.905748 | -2.739501 |
| NM_178399 | 3110035E14Rik | n/a | -3.469235 | -3.743542 | -2.501977 |
| NM_023727 | 3322402L07Rik | n/a | -3.738818 | -3.502052 | -3.72381 |
| NM_177377 | 4833436C18Rik | n/a | -3.807853 | -4.161158 | -4.367965 |
| BC050813 | 4921511C04Rik | n/a | -3.382215 | -3.066267 | -2.849396 |
| ENSMUST00000051862 | 4922501L14Rik | n/a | -3.397848 | -4.007188 | -2.804533 |
| AK132588 | 4930451C15Rik | n/a | -4.085425 | -2.909795 | -3.955416 |
| AK220398 | 4930528G09Rik | n/a | -3.090776 | -3.101435 | -3.878962 |
| AK016265 | 4930570G19Rik | n/a | -3.749898 | -2.953431 | -2.663766 |
| NM_001081025 | 4932425I24Rik | n/a | -2.728159 | -2.775713 | -3.530352 |
| AK037440 | 4933407L21Rik | n/a | -4.143985 | -3.617943 | -3.117621 |
| ENSMUST00000113976 | 5430427O19Rik | n/a | -4.909336 | -2.750446 | -5.05011 |
| NM_177656 | 6820408C15Rik | n/a | -4.499092 | -3.101282 | -4.506904 |
| AK047163 | 8030453O22Rik | n/a | -3.377854 | -2.867892 | -3.323804 |
| AK020388 | 9330179D12Rik | n/a | -2.758953 | -2.501908 | -2.982509 |
| NM_177713 | 9830130M13Rik | n/a | -3.359594 | -2.645936 | -3.670696 |
| A_51_P144143 | A_51_P144143 | n/a | -5.378137 | -3.12338 | -5.704936 |
| A_51_P402908 | A_51_P402908 | n/a | -4.284056 | -4.375517 | -4.835746 |
| NM_178785 | A430107D22Rik | n/a | -4.493195 | -2.467064 | -5.17598 |
| AK142678 | A530040E14Rik | n/a | -3.435467 | -2.665715 | -3.590056 |
| AK044281 | A930005G22Rik | n/a | -3.319893 | -4.939803 | -2.688274 |
| NM_025659 | Abi3 | ABI gene family, member 3 | -3.224 | -2.762312 | -3.872538 |
| NM_009608 | Actc1 | actin, | -3.410102 | -3.936999 | -2.469893 |
| NM_001037724 | Adcy7 | adenylate cyclase 7 | -3.181097 | -3.163672 | -4.053072 |
| NM_007406 | Adcy7 | adenylate cyclase 7 | -3.513073 | -2.76253 | -4.376145 |
| NM_134158 | AF251705 | n/a | -4.67647 | -3.240988 | -5.150808 |
| NM_178755 | Agbl2 | ATP/GTP binding protein-like 2 | -3.507197 | -3.135524 | -2.636236 |
| NM_007428 | Agt | angiotensinogen | -3.522087 | -2.962588 | -2.857009 |
| NM_019467 | Aif1 | allograft inflammatory factor 1 | -5.084646 | -4.55845 | -5.919435 |
| AK012436 | AK012436 | n/a | -2.505563 | -3.629252 | -4.452914 |
| AK045879 | AK045879 | n/a | -3.092294 | -2.719877 | -2.498107 |
| AK051526 | AK051526 | n/a | -2.421222 | -2.676934 | -2.300208 |
| AK051578 | AK051578 | n/a | -2.733431 | -2.51322 | -2.722278 |
| AK081101 | AK081101 | n/a | -3.305683 | -2.616533 | -2.45768 |
| AK087644 | AK087644 | n/a | -3.130993 | -2.826528 | -3.400179 |
| AK029555 | Ak7 | n/a | -3.667312 | -2.734175 | -3.865814 |
| NM_001033785 | Akap14 | A kinase anchor protein 14 | -6.287872 | -4.499892 | -6.425938 |
| NM_009663 | Alox5ap | arachidonate 5-lipoxygenase activating protein | -4.007135 | -3.750931 | -4.488045 |
| NM_019456 | Apbb1ip | amyloid beta interacting protein | -4.265389 | -3.24781 | -3.392995 |
| NM_009700 | Aqp4 | aquaporin 4 | -3.362429 | -3.295047 | -2.367738 |
| NM_007482 | Arg1 | arginase 1 | -7.064551 | -5.313859 | -7.858431 |
| NM_001037727 | Arhgap25 | Rho GTPase activating protein 25 | -4.250387 | -4.735373 | -4.935283 |
| NM_001005508 | Arhgap30 | Rho GTPase activating protein 30 | -5.865139 | -3.898739 | -6.663335 |
| NM_007486 | Arhgdib | Rho, GDP dissociation inhibitor | -5.207379 | -4.485348 | -5.275101 |
| NM_008113 | Arhgdig | Rho GDP dissociation inhibitor | -2.651533 | -2.437841 | -2.769011 |
| NM_177337 | Arl11 | ADP-ribosylation factor-like 11 | -5.127924 | -3.414755 | -5.665716 |
| NM_175406 | Atp6v0d2 | ATPase, H+ transporting, lysosomal | -3.620294 | -2.847304 | -3.958842 |
| NM_177629 | AU021034 | n/a | -4.701036 | -3.291485 | -5.155908 |
| NM_153539 | B830045N13Rik | n/a | -2.920412 | -2.915722 | -2.755881 |
| NM_016767 | Batf | basic leucine zipper transcription factor | -3.924765 | -3.016921 | -4.443404 |
| NM_130452 | Bbox1 | butyrobetaine | -5.429326 | -3.64066 | -4.029648 |
| AK030294 | BC007180 | n/a | -3.892826 | -3.358647 | -2.784677 |
| NM_001025575 | BC026782 | n/a | -4.820662 | -3.515206 | -4.331037 |
| CB599241 | BC028528 | n/a | -3.186536 | -3.426271 | -2.935449 |
| NM_182783 | BC030183 | n/a | -4.445381 | -2.619415 | -4.32571 |
| NM_001081369 | BC038167 | n/a | -3.105511 | -3.59823 | -2.728743 |
| NM_183187 | BC055107 | n/a | -3.13449 | -2.911654 | -2.496557 |
| NM_007534 | Bcl2a1b | B-cell leukemia/lymphoma 2 related protein | -6.907932 | -4.731956 | -7.581713 |
| NM_007535 | Bcl2a1c | B-cell leukemia/lymphoma 2 related protein | -5.473983 | -4.394242 | -6.033804 |
| NM_008528 | Blnk | B-cell linker | -5.198132 | -3.788505 | -4.844283 |
| NM_013482 | Btk | Bruton agammaglobulinemia tyrosine kinase | -5.295405 | -3.423217 | -5.843626 |
| NM_175219 | C130026I21Rik | n/a | -5.640728 | -3.399327 | -6.231057 |
| NM_007572 | C1qa | complement component 1, q subcomponent, alpha | -6.813727 | -5.392683 | -7.328888 |
| NM_009777 | C1qb | complement component 1, q subcomponent, beta | -4.522381 | -2.883992 | -4.95851 |
| NM_007574 | C1qc | complement component 1, q subcomponent, C chain | -8.390108 | -6.60598 | -8.397368 |
| AK049932 | C230072F16Rik | n/a | -4.028604 | -2.441346 | -3.040509 |
| NM_009779 | C3ar1 | complement component 3a receptor 1 | -4.416981 | -2.631626 | -4.90328 |
| AK050399 | C730043O17 | n/a | -4.208199 | -3.855324 | -2.867432 |
| NM_138304 | Calml4 | calmodulin-like 4 | -4.083333 | -4.155225 | -3.733327 |
| NM_007601 | Capn3 | calpain 3 | -3.726151 | -2.633238 | -4.68085 |
| NM_009807 | Casp1 | caspase 1 | -4.202378 | -3.883195 | -2.522623 |
| AK029544 | Ccdc81 | n/a | -2.430428 | -2.684917 | -2.68386 |
| NM_011331 | Ccl12 | chemokine 12 | -6.431568 | -5.375035 | -7.091487 |
| NM_019577 | Ccl24 | chemokine 24 | -4.677014 | -3.141224 | -5.1037 |
| NM_011337 | Ccl3 | chemokine 3 | -6.312389 | -4.785546 | -6.658253 |
| NM_013652 | Ccl4 | chemokine 4 | -4.179942 | -3.525269 | -4.84545 |
| NM_009139 | Ccl6 | chemokine 6 | -7.102498 | -4.191677 | -7.205515 |
| NM_011338 | Ccl9 | chemokine ligand 9 | -5.209047 | -3.987423 | -6.652531 |
| NM_009917 | Ccr5 | chemokine receptor 5 | -5.262733 | -3.629182 | -5.804156 |
| NM_009841 | Cd14 | CD14 antigen | -4.692387 | -2.96426 | -4.622991 |
| AK089613 | Cd180 | n/a | -5.24961 | -4.186747 | -5.77591 |
| NM_008533 | Cd180 | CD180 antigen | -4.319906 | -2.772031 | -4.758835 |
| NM_021325 | Cd200r1 | CD200 receptor | -4.324514 | -3.006785 | -4.87754 |
| NM_145634 | Cd300lf | CD300 antigen | -4.254719 | -4.430781 | -6.376813 |
| NM_007646 | Cd38 | CD38 antigen | -4.012849 | -3.029609 | -3.932372 |
| NM_013706 | Cd52 | CD52 antigen | -4.174276 | -3.780236 | -5.209448 |
| NM_007651 | Cd53 | CD53 antigen | -6.811436 | -4.458014 | -7.514707 |
| NM_013489 | Cd84 | CD84 antigen | -7.043743 | -4.868154 | -7.79964 |
| NM_019388 | Cd86 | CD86 antigen | -4.182466 | -2.722914 | -4.678628 |
| NM_007675 | Ceacam10 | CEA-related cell adhesion molecule 10 | -3.711492 | -3.076723 | -3.938756 |
| M12660 | Cfh | Complement (h) | -5.675797 | -3.799162 | -4.320397 |
| NM_009888 | Cfh | complement component factor h | -6.405655 | -5.448569 | -4.679741 |
| NM_009890 | Ch25h | cholesterol 25-hydroxylase | -4.086209 | -3.638734 | -4.133486 |
| NM_023186 | Chia | musculus chitinase | -3.965653 | -2.715549 | -3.752518 |
| NM_009899 | Clca1 | chloride channel calcium activated 1 | -3.344432 | -2.532229 | -3.943818 |
| NM_008770 | Cldn11 | claudin 11 | -2.463897 | -2.457271 | -2.771593 |
| NM_153197 | Clec4a3 | C-type lectin domain family 4, member a3 | -3.541476 | -2.52563 | -3.852658 |
| NM_010819 | Clec4d | C-type lectin 4d | -6.358351 | -4.43909 | -7.010943 |
| NM_020001 | Clec4n | C-type lectin 4n | -4.571344 | -3.152974 | -5.374429 |
| NM_001038604 | Clec5a | C-type lectin 5a | -4.187126 | -2.525178 | -4.579767 |
| NM_021364 | Clec5a | C-type lectin 5a | -3.554329 | -2.894998 | -3.904722 |
| NM_020008 | Clec7a | C-type lectin 7a | -6.81585 | -5.138442 | -7.29962 |
| NM_009898 | Coro1a | coronin, actin binding protein 1A | -2.444602 | -3.359835 | -2.957424 |
| NM_053250 | Crip3 | cysteine-rich protein 3 | -2.570562 | -2.857404 | -3.255943 |
| NM_001037859 | Csf1r | colony stimulating factor 1 receptor | -5.058815 | -3.150239 | -5.659597 |
| NM_007781 | Csf2rb2 | colony stimulating factor 2 receptor | -5.501829 | -3.629983 | -6.094971 |
| NM_007782 | Csf3r | colony stimulating factor 3 receptor | -4.096705 | -3.617494 | -4.490188 |
| NM_007796 | Ctla2a | cytotoxic T lymphocyte-associated protein 2 alpha | -7.502928 | -4.852581 | -7.181286 |
| BC005432 | Ctse | cathepsin E | -3.949847 | -2.873202 | -4.689999 |
| NM_021281 | Ctss | cathepsin S | -8.127447 | -5.084961 | -7.78048 |
| NM_009987 | Cx3cr1 | chemokine (C-X3-C) receptor 1 | -4.583337 | -3.388824 | -5.047336 |
| NM_007807 | Cybb | cytochrome b-245 | -4.249325 | -2.546022 | -4.61226 |
| NM_021476 | Cysltr1 | cysteinyl leukotriene receptor 1 | -5.026319 | -3.746545 | -4.743793 |
| NM_001037905 | Dab2 | disabled homolog 2 | -2.975249 | -3.075353 | -2.732673 |
| NM_007833 | Dcn | decorin | -3.473063 | -3.264222 | -2.431203 |
| NM_027185 | Def6 | differentially expressed in FDCP 6 | -3.147256 | -2.720934 | -3.583977 |
| NM_011303 | Dhrs3 | dehydrogenase/reductase 3 | -3.419303 | -3.316231 | -2.653796 |
| NM_028618 | Dmkn | musculus dermokine | -4.169081 | -3.509888 | -4.598388 |
| NM_153527 | Dnajb13 | DnaJ B13 | -2.863804 | -2.574539 | -2.567279 |
| NM_033374 | Dock2 | dedicator of cyto-  kinesis 2 | -4.162659 | -2.568384 | -4.539095 |
| AK018051 | Dock8 | n/a | -3.741581 | -2.7557 | -4.38855 |
| NM_028785 | Dock8 | dedicator of cytokinesis 8 | -3.656162 | -2.667759 | -4.332978 |
| NM_010071 | Dok2 | docking protein 2 | -3.319974 | -3.501423 | -3.794949 |
| XM_001474855 | E030010N08Rik | n/a | -4.920354 | -4.466746 | -5.7863 |
| XM_149293 | E030011K20Rik | n/a | -5.37312 | -4.559248 | -5.926393 |
| NM_183031 | Ebi2 | Epstein-Barr virus induced gene 2 | -6.689966 | -3.972757 | -6.867738 |
| NM_001012324 | Ecm2 | extracellular matrix protein 2 | -4.798339 | -3.135344 | -4.514461 |
| AK042211 | Ednra | n/a | -2.495079 | -2.86785 | -2.67112 |
| NM_001029977 | EG214403 | n/a | -4.515497 | -3.125656 | -4.946353 |
| AK045784 | EG328314 | n/a | -4.31536 | -3.368168 | -3.927997 |
| XM_619025 | EG544888 | n/a | -3.127502 | -2.517358 | -2.893533 |
| NM_133918 | Emilin1 | elastin microfibril interfacer 1 | -3.412166 | -3.175232 | -3.240146 |
| NM_010129 | Emp3 | epithelial membrane protein 3 | -2.649959 | -2.657377 | -2.405736 |
| NM_010130 | Emr1 | EGF-like module containing, mucin-like, hormone receptor-like sequence 1 | -5.647077 | -4.517735 | -6.629197 |
| AK165234 | ENSMUSG00000074658 | n/a | -3.996857 | -2.744754 | -2.884055 |
| ENSMUST00000021884 | ENSMUST00000021884 | n/a | -6.962581 | -4.659783 | -7.456932 |
| ENSMUST00000046994 | ENSMUST00000046994 | n/a | -5.77424 | -3.360929 | -6.360425 |
| ENSMUST00000063955 | ENSMUST00000063955 | n/a | -5.425146 | -3.863835 | -3.975713 |
| ENSMUST00000065383 | ENSMUST00000065383 | n/a | -4.029658 | -2.658602 | -4.216178 |
| ENSMUST00000116243 | ENSMUST00000116243 | n/a | -2.638696 | -2.466985 | -2.508661 |
| NM_009848 | Entpd1 | ectonucleoside triphosphate diphosphohydrolase 1 | -3.260203 | -2.491118 | -2.523195 |
| NM_029495 | Epsti1 | epithelial stromal interaction 1 | -4.967463 | -3.834909 | -5.143561 |
| NM_030711 | Erap1 | endoplasmic reticulum aminopeptidase 1 | -3.006964 | -2.612904 | -2.366862 |
| NM_001033711 | Evi2a | ecotropic viral integration site 2a | -6.67977 | -5.0628 | -7.370986 |
| NM_146023 | Evi2b | ecotropic viral integration site 2b | -3.790927 | -2.663676 | -4.281347 |
| NM_028784 | F13a1 | coagulation factor XIII, A1 subunit | -8.227017 | -6.933259 | -9.085986 |
| NM_177010 | F630003A18Rik | n/a | -4.101411 | -2.724347 | -4.520317 |
| J05020 | Fcer1g | mast cell high affinity IgE receptor (Fc-epsilon-RI) gamma | -6.043391 | -5.618241 | -7.400475 |
| NM_010185 | Fcer1g | Fc receptor, IgE, high affinity I, gamma | -5.229211 | -3.859194 | -5.976507 |
| AF143181 | Fcgr1 | AB/H (Biozzi) high affinity immunoglobulin gamma Fc receptor I | -5.85964 | -4.45684 | -7.014176 |
| NM_010186 | Fcgr1 | Fc receptor, IgG, high affinity I | -6.266976 | -5.293864 | -7.496499 |
| NM_001077189 | Fcgr2b | Fc receptor, IgG, low affinity IIb | -7.500785 | -5.28654 | -7.709768 |
| NM_010187 | Fcgr2b | Fc receptor, IgG, low affinity IIb | -7.450028 | -5.784508 | -7.780285 |
| NM_010188 | Fcgr3 | Fc receptor, IgG, low affinity III | -3.788314 | -4.008386 | -4.547426 |
| NM_007995 | Fcna | ficolin A | -6.756782 | -4.268049 | -7.076946 |
| NM_030707 | Fcrls | Fc receptor-like S, scavenger receptor | -5.490426 | -4.414123 | -5.971736 |
| NM_153795 | Fermt3 | fermitin 3 | -4.181897 | -3.07806 | -5.862765 |
| NM_010194 | Fes | feline sarcoma oncogene | -3.626408 | -3.443724 | -4.320647 |
| NM_008026 | Fli1 | Friend leukemia integration 1 | -5.417967 | -4.881602 | -5.313263 |
| NM_008034 | Folr1 | folate receptor 1 | -2.936964 | -3.081715 | -2.57197 |
| NM_008035 | Folr2 | folate receptor 2 | -5.462502 | -3.87553 | -5.698249 |
| NM_011815 | Fyb | FYN binding protein | -5.01307 | -3.596878 | -5.601909 |
| NM_001033416 | Gal3st4 | galactose-3-O-sulfotransferase 4 | -4.486493 | -2.810542 | -2.773009 |
| NM_025961 | Gatm | glycine amidinotransferase | -3.705667 | -3.100866 | -3.415492 |
| AK140151 | Gfap | glial fibrillary acidic protein | -3.9381 | -4.167657 | -2.742436 |
| K01347 | Gfap | glial fibrillary acidic protein | -4.945211 | -5.191688 | -4.23451 |
| NM_028608 | Glipr1 | GLI pathogenesis-related 1 | -3.40238 | -3.368645 | -3.465521 |
| NM_008134 | Glycam1 | glycosylation dependent cell adhesion molecule 1 | -5.0623 | -3.210183 | -3.917479 |
| ENSMUST00000036088 | Gngt2 | Guanine nucleotide-binding protein | -3.079299 | -2.824379 | -3.364483 |
| NM_053110 | Gpnmb | glycoprotein nmb | -3.90594 | -3.116802 | -4.105011 |
| NM_001081220 | Gpr179 | G protein-coupled receptor 179 | -2.665806 | -2.821929 | -3.505007 |
| NM_011823 | Gpr34 | G protein-coupled receptor 34 | -6.543395 | -5.242422 | -7.230206 |
| NM_134438 | Gpr37l1 | G protein-coupled receptor 37-like 1 | -2.885945 | -3.181821 | -2.582242 |
| NM_008152 | Gpr65 | G-protein coupled receptor 65 | -5.178121 | -4.356035 | -6.223694 |
| NM_030720 | Gpr84 | G protein-coupled receptor 84 | -3.928403 | -2.907253 | -4.369329 |
| NM_027817 | Grap | GRB2-related adaptor protein | -6.163979 | -4.198642 | -6.397963 |
| NM_010368 | Gusb | glucuronidase, beta | -2.807345 | -3.199462 | -2.991195 |
| NM_029000 | Gvin1 | GTPase, very large interferon inducible 1 transcript variant A, | -3.329788 | -2.509942 | -2.498905 |
| NM_010406 | Hc | hemolytic complement | -4.218012 | -3.271274 | -3.432298 |
| NM_008225 | Hcls1 | hematopoietic cell specific Lyn substrate 1 | -4.853503 | -3.598431 | -5.45483 |
| NM_008230 | Hdc | histidine decarboxylase | -4.035624 | -3.898901 | -3.925559 |
| NM_175189 | Hepacam | hepatocyte cell adhesion molecule | -4.059906 | -2.528455 | -3.044599 |
| NM_001033245 | Hk3 | hexokinase 3 | -4.39574 | -3.041861 | -5.156963 |
| NM_008278 | Hpgd | hydroxyprostaglandin dehydrogenase | -3.287338 | -4.00322 | -3.029562 |
| NM_008330 | Ifi47 | interferon gamma inducible protein 47 | -3.601032 | -2.939225 | -2.322289 |
| NM_001033632 | Ifitm6 | interferon induced transmembrane protein 6 | -3.371054 | -3.285039 | -2.398299 |
| NM_010517 | Igfbp4 | insulin-like growth factor binding protein 4 | -3.901259 | -3.125671 | -3.693203 |
| NM_010518 | Igfbp5 | insulin-like growth factor binding protein 5 | -3.978178 | -3.888796 | -3.577553 |
| NM_030691 | Igsf6 | immunoglobulin superfamily, member 6 | -5.121016 | -3.174042 | -5.03646 |
| NM_001025597 | Ikzf1 | IKAROS family zinc finger 1 | -4.832989 | -2.718832 | -4.750581 |
| NM_008348 | Il10ra | interleukin 10 receptor, alpha | -3.609371 | -2.489731 | -4.621238 |
| AK085780 | Il1rl2 | n/a | -3.639094 | -3.535626 | -4.005118 |
| NM_010566 | Inpp5d | inositol polyphosphate-5-phosphatase D | -6.983193 | -5.724068 | -6.900995 |
| NM_012043 | Islr | immunoglobulin superfamily containing leucine-rich repeat | -3.266493 | -3.283465 | -2.731636 |
| AK156251 | Itgal | n/a | -2.38014 | -3.094225 | -2.696869 |
| NM_001082960 | Itgam | integrin alpha M | -5.975072 | -3.706209 | -6.597456 |
| NM_008401 | Itgam | integrin alpha M | -4.638333 | -3.079581 | -5.076695 |
| NM_021334 | Itgax | integrin alpha X | -4.220661 | -2.836908 | -4.60862 |
| NM_008404 | Itgb2 | integrin beta 2 | -4.1287 | -2.642268 | -4.855333 |
| NM_008407 | Itih3 | inter-alpha trypsin inhibitor, heavy chain 3 | -4.912954 | -4.249902 | -3.843797 |
| NM_010604 | Kcnj16 | potassium inwardly-rectifying channel J16 | -4.042605 | -2.63606 | -3.415076 |
| NM_001033525 | Kcnk6 | potassium inwardly-rectifying channel K6 | -3.36178 | -3.540417 | -4.368535 |
| NM_183390 | Klhl6 | kelch-like 6 | -5.811666 | -3.287841 | -4.72591 |
| NM_178611 | Lair1 | leukocyte-associated Ig-like receptor 1 | -4.296595 | -2.777645 | -4.512866 |
| NM_010686 | Laptm5 | lysosomal-associated protein transmembrane 5 | -5.822534 | -3.933029 | -6.422129 |
| NM_020044 | Lat2 | linker for activation of T cells 2 | -3.345986 | -2.705562 | -4.032652 |
| NM_008879 | Lcp1 | lymphocyte cytosolic protein 1 | -3.840665 | -3.285678 | -4.837083 |
| NM_010696 | Lcp2 | lymphocyte cytosolic protein 2 | -5.305868 | -3.557639 | -4.715515 |
| NM_010701 | Lect1 | leukocyte cell derived chemotaxin 1 | -3.491595 | -4.25423 | -3.322486 |
| AK035873 | Lgi1 | n/a | -4.153348 | -2.788774 | -3.586458 |
| NM_020278 | Lgi1 | leucine-rich repeat LGI | -3.940817 | -2.803975 | -3.448203 |
| NM_001081231 | Lhfpl3 | lipoma HMGIC fusion partner-like 3 | -2.48421 | -3.30297 | -2.317149 |
| NM_011095 | Lilrb3 | leukocyte immunoglobulin-like receptor, b3 | -5.826765 | -4.080136 | -6.791327 |
| NM_013532 | Lilrb4 | leukocyte immunoglobulin-like receptor, b4 | -4.826777 | -3.154039 | -5.304999 |
| NM_008505 | Lmo2 | LIM domain only 2 | -3.589266 | -2.929591 | -3.421467 |
| ENSMUST00000050623 | LOC100038947 | SIRP beta 1 cell surface protein. | -4.17381 | -3.532163 | -4.49562 |
| NM_008509 | Lpl | lipoprotein lipase | -2.823042 | -2.552068 | -2.724467 |
| NM_134152 | Lpxn | leupaxin | -2.50534 | -3.461089 | -2.86819 |
| NM_027452 | Lrfn2 | leucine rich repeat and fibronectin type III domain containing 2 | -2.487111 | -3.775985 | -3.054303 |
| NM_008511 | Lrmp | lymphoid-restricted membrane protein | -5.011663 | -3.662842 | -5.38749 |
| NM_013588 | Lrrc23 | leucine rich repeat containing 23 | -3.159191 | -3.283085 | -3.018675 |
| NM_146069 | Lrrc33 | leucine rich repeat containing 33 | -7.32186 | -5.309965 | -8.287866 |
| NM_138682 | Lrrc4 | leucine rich repeat containing 4 | -3.469831 | -3.985754 | -2.63756 |
| NM_019391 | Lsp1 | lymphocyte specific 1 | -5.242782 | -3.062301 | -4.003013 |
| NM_008521 | Ltc4s | leukotriene C4 synthase | -4.934366 | -4.306168 | -5.973964 |
| NM_008524 | Lum | lumican | -3.206104 | -3.313799 | -2.635974 |
| NM_010745 | Ly86 | lymphocyte antigen 86 | -8.223199 | -5.835727 | -8.458595 |
| NM_008535 | Lyl1 | lymphoblastomic leukemia 1 | -5.543963 | -4.370586 | -5.908728 |
| NM_010747 | Lyn | Yamaguchi sarcoma viral oncogene homolog | -3.125538 | -3.241994 | -2.355947 |
| NM_053247 | Lyve1 | lymphatic vessel endothelial hyaluronan receptor 1 | -7.255861 | -5.597509 | -8.144077 |
| NM_013590 | Lyz1 | lysozyme 1 | -7.231465 | -3.985984 | -7.314802 |
| AK159276 | Lyz2 | n/a | -6.417783 | -3.531242 | -6.498047 |
| NM_017372 | Lyz2 | lysozyme 2 | -6.306161 | -3.424022 | -6.061087 |
| NM_174857 | Mamdc2 | MAM domain containing 2 | -3.853431 | -3.68972 | -2.907719 |
| NM_010777 | Mbp | myelin basic protein | -3.172241 | -3.608687 | -4.176538 |
| NM_010796 | Mgl1 | macrophage galactose N-acetyl-galactosamine specific lectin 1 | -4.879103 | -4.496239 | -5.581591 |
| NM_133241 | Mlc1 | megalencephalic leukoencephalopathy with subcortical cysts 1 homolog | -2.816358 | -2.777546 | -2.298508 |
| AK083103 | Mobp | myelin-associated oligodendrocytic basic protein | -3.420239 | -3.403328 | -4.338669 |
| NM_029112 | Morn3 | MORN repeat 3 | -3.816332 | -2.624669 | -4.538754 |
| NM_010821 | Mpeg1 | macrophage expressed gene 1 | -5.052138 | -3.987158 | -4.519777 |
| NM_008625 | Mrc1 | mannose receptor, C1 | -7.587681 | -5.968291 | -8.964005 |
| NM_027209 | Ms4a6b | membrane-spanning 4A6B | -7.298997 | -5.698119 | -7.035282 |
| NM_028595 | Ms4a6c | membrane-spanning 4a6C | -4.271428 | -3.041654 | -5.156422 |
| AB026047 | Ms4a6d | n/a | -5.3111 | -4.036846 | -5.383409 |
| NM_026835 | Ms4a6d | membrane-spanning 4A6d | -6.454804 | -4.674803 | -6.268475 |
| NM_001025610 | Ms4a7 | membrane-spanning 4A7 | -5.649113 | -4.101024 | -6.155874 |
| NM_031195 | Msr1 | macrophage scavenger receptor | -7.1062 | -4.854503 | -7.638114 |
| AK142590 | Muc15 | n/a | -4.413449 | -2.86991 | -3.251315 |
| AK036518 | Myb | n/a | -2.956383 | -3.596615 | -4.248353 |
| NM_010848 | Myb | myeloblastosis oncogene | -2.857088 | -3.061433 | -3.279093 |
| NM_053214 | Myo1f | myosin IF | -4.851851 | -3.190225 | -5.488946 |
| NM_178440 | Myo1g | myosin IG | -4.489649 | -3.721153 | -6.173399 |
| NM_010872 | Naip2 | NLR family, apoptosis inhibitory protein 2 | -5.183386 | -3.251379 | -5.304294 |
| NM_010870 | Naip5 | NLR family, apoptosis inhibitory protein 5 | -4.169328 | -3.066811 | -4.339525 |
| NAP037326-1 | NAP037326-1 | n/a | -7.09504 | -4.983039 | -7.825792 |
| NAP050063-1 | NAP050063-1 | n/a | -3.163133 | -2.90084 | -2.851876 |
| NM_010876 | Ncf1 | neutrophil cytosolic factor 1 | -4.945126 | -3.207699 | -5.361816 |
| NM_010877 | Ncf2 | neutrophil cytosolic factor 2 | -4.046926 | -4.177344 | -4.532548 |
| NM_008677 | Ncf4 | neutrophil cytosolic factor 4 | -3.213022 | -4.237731 | -4.330289 |
| NM_153505 | Nckap1l | NCK associated protein 1 like | -3.856758 | -3.480017 | -4.536695 |
| NM_010883 | Ndph | Norrie disease homolog | -3.853132 | -3.630245 | -2.704853 |
| AK031027 | Neil3 | n/a | -3.009535 | -2.462699 | -3.280013 |
| NM_146208 | Neil3 | nei like 3 | -5.156006 | -2.946849 | -5.512415 |
| NM_177898 | Nek5 | NIMA (never in mitosis gene a)-related expressed kinase 5 | -2.576629 | -4.072329 | -2.418503 |
| NM_145211 | Oas1a | 2'-5' oligoadenylate synthetase 1A | -2.842885 | -3.106841 | -2.386648 |
| NM_021879 | Oca2 | oculocutaneous albinism II | -3.185646 | -2.793122 | -2.487453 |
| NM_016968 | Olig1 | oligodendrocyte transcription factor 1 | -3.460601 | -3.502235 | -2.776144 |
| NM_001081957 | OTTMUSG00000000971 | n/a | -6.262183 | -4.384078 | -6.922283 |
| AK054195 | P2rx6 | purinergic receptor P2X-like 1, orphan receptor | -5.378743 | -3.330684 | -4.552406 |
| NM_027571 | P2ry12 | purinergic receptor P2Y, G-protein coupled 12 | -5.470735 | -3.756282 | -6.419691 |
| NM_028808 | P2ry13 | purinergic receptor P2Y, G-protein coupled 13 | -5.872969 | -4.526897 | -6.872089 |
| NM_183168 | P2ry6 | pyrimidinergic receptor P2Y, G-protein coupled, 6 | -3.359177 | -5.056428 | -4.006846 |
| NM_022321 | Parvg | parvin, gamma | -4.13916 | -2.550353 | -4.909339 |
| NM_019932 | Pf4 | platelet factor 4 | -8.502647 | -6.364641 | -8.40832 |
| NM_172603 | Phf11 | PHD finger protein 11 | -3.204834 | -2.441918 | -4.163301 |
| NM_011079 | Phkg1 | phosphorylase kinase gamma 1 | -4.355609 | -2.563225 | -4.267741 |
| NM_020272 | Pik3cg | phosphoinositide-3-kinase, catalytic, gamma polypeptide | -5.325407 | -3.486801 | -5.263911 |
| NM_153510 | Pilra | paired immunoglobin-like type 2 receptor alpha | -5.08774 | -2.925644 | -5.611105 |
| NM_011089 | Pira2 | paired-Ig-like receptor A2 | -2.77611 | -2.53658 | -3.036547 |
| NM_008873 | Plau | plasminogen activator, urokinase | -2.408207 | -3.203627 | -2.319039 |
| NM_178911 | Pld4 | phospholipase D family, member 4 | -5.197051 | -3.544053 | -5.224943 |
| NM_019549 | Plek | pleckstrin | -5.30585 | -4.245283 | -5.945781 |
| NM_011123 | Plp1 | proteolipid protein | -2.552889 | -2.463656 | -3.205991 |
| NM_173006 | Pon3 | paraoxonase 3 | -4.224824 | -4.3624 | -3.742675 |
| NM_023785 | Ppbp | pro-platelet basic protein | -3.420449 | -3.04892 | -3.739992 |
| XM_357002 | Psma8 | proteasome subunit, alpha type, 8 | -3.642968 | -2.850901 | -3.357178 |
| NM_019455 | Ptgds2 | prostaglandin D2 synthase 2, | -5.527218 | -3.717801 | -4.971602 |
| NM_001077705 | Ptpn6 | protein tyrosine phosphatase, non-receptor type 6 | -2.522422 | -4.064257 | -3.56823 |
| NM_011210 | Ptprc | protein tyrosine phosphatase, receptor type, C | -5.696352 | -4.80847 | -5.988889 |
| NM_023258 | Pycard | PYD and CARD domain containing | -4.711758 | -3.330383 | -4.353027 |
| NM_009008 | Rac2 | RAS-related C3 botulinum substrate 2 | -5.54694 | -4.006476 | -6.006022 |
| AK122234 | Rapgef5 | mRNA for mKIAA0277 protein | -2.965091 | -2.519604 | -3.688566 |
| NM_207246 | Rasgrp3 | RAS, guanyl releasing protein 3 | -4.192837 | -4.820409 | -3.759239 |
| AK152971 | Rbm47 | n/a | -4.09154 | -2.535511 | -4.545696 |
| NM_139065 | Rbm47 | RNA binding motif protein 47 | -4.085161 | -2.740037 | -4.622636 |
| NM_134083 | Rcbtb2 | regulator of chromosome condensation (RCC1) and BTB (POZ) domain containing protein 2 | -3.210569 | -3.879353 | -2.347903 |
| AK030934 | Rcsd1 | n/a | -2.984488 | -2.674617 | -3.001713 |
| NM_178593 | Rcsd1 | RCSD domain containing 1 | -2.769938 | -2.964132 | -2.939149 |
| NM_015811 | Rgs1 | regulator of G-protein signaling 1 | -4.884408 | -3.240018 | -5.362265 |
| XM_891200 | Rgs22 | regulator of G-protein signalling 22 | -3.211301 | -2.633903 | -3.314688 |
| NM_028724 | Rin2 | Ras and Rab interactor 2 | -2.939345 | -2.886381 | -2.461048 |
| NM_011882 | Rnasel | ribonuclease L (2', 5'-oligoisoadenylate synthetase-dependent) | -2.433196 | -2.800993 | -2.568032 |
| NM_009115 | S100b | S100 protein, beta polypeptide, neural | -3.467269 | -2.629884 | -2.562002 |
| NM_023380 | Samsn1 | SAM domain, SH3 domain and nuclear localization signals, 1 | -6.611247 | -4.638147 | -7.311465 |
| NM_028773 | Sash3 | SAM and SH3 domain containing 3 | -5.540879 | -3.88377 | -6.171519 |
| NM_183216 | Scd4 | stearoyl-coenzyme A desaturase 4 | -2.656669 | -3.640321 | -2.971855 |
| NM_009136 | Scrg1 | scrapie responsive gene 1 | -3.085755 | -3.707437 | -2.472188 |
| NM_009252 | Serpina3n | serine (or cysteine) peptidase inhibitor,A3N | -3.963182 | -3.724727 | -3.921177 |
| NM_198028 | Serpinb10 | serine (or cysteine) peptidase inhibitor,B10 | -6.304669 | -4.612925 | -5.576966 |
| NM_007547 | Sirpa | signal-regulatory protein alpha | -3.043413 | -2.830379 | -2.560106 |
| NM_029612 | Slamf9 | SLAM family member 9 | -3.532031 | -3.345799 | -4.315012 |
| NM_023044 | Slc15a3 | solute carrier family 15, member 3 | -4.987361 | -3.414204 | -5.794747 |
| NM_001081048 | Slc25a18 | solute carrier family 25 (mitochondrial carrier), member 18 (Slc25a18) | -5.489601 | -3.920821 | -5.063247 |
| NM_001012305 | Slc39a12 | solute carrier family 39 (zinc transporter), member 12 | -5.088599 | -4.123195 | -3.954507 |
| NM_030687 | Slco1a4 | solute carrier organic anion transporter 1a4 | -4.738735 | -3.971918 | -3.259998 |
| NM_021471 | Slco1c1 | solute carrier organic anion transporter 1c1 | -3.589773 | -2.635303 | -2.38216 |
| NM_175316 | Slco2b1 | solute carrier organic anion transporter 2b1 | -6.168247 | -5.367339 | -6.53467 |
| NM_011408 | Slfn2 | schlafen 2 | -5.339555 | -3.28076 | -4.708444 |
| NM_011414 | Slpi | secretory leukocyte peptidase inhibitor | -2.782401 | -2.517616 | -3.613586 |
| NM_175397 | Sp110 | Sp110 nuclear body protein | -6.857047 | -5.025606 | -6.308325 |
| NM_145584 | Spon1 | spondin 1 | -2.947337 | -2.639711 | -2.780371 |
| NM_138672 | Stab1 | stabilin | -5.474678 | -3.876809 | -6.238565 |
| NM_133670 | Sult1a1 | sulfotransferase family 1A, phenol-preferring 1 | -3.241554 | -2.622908 | -2.52177 |
| NM_011539 | Tbxas1 | thromboxane A synthase 1, platelet | -6.847042 | -4.288697 | -7.113836 |
| TC1610785 | TC1610785 | Grb10 interacting protein | -3.959524 | -3.919971 | -2.993725 |
| TC1637884 | TC1637884 | CTLA-2-beta protein precursor | -6.863437 | -5.592646 | -8.049403 |
| TC1658525 | TC1658525 | n/a | -5.028323 | -4.681561 | -5.122603 |
| TC1671899 | TC1671899 | n/a | -5.767125 | -3.709679 | -6.015833 |
| NM_031198 | Tcfec | transcription factor EC | -3.782996 | -2.596254 | -4.398256 |
| NM_013688 | Tcte1 | t-complex-associated testis expressed 1 | -4.173816 | -3.658426 | -4.359425 |
| NM_009369 | Tgfbi | transforming growth factor, beta induced | -3.785915 | -3.736759 | -4.133022 |
| NM_178759 | Timd4 | T-cell immunoglobulin and mucin domain containing 4 | -2.983656 | -2.73802 | -3.317414 |
| NM_009387 | Tk1 | thymidine kinase 1 | -3.304224 | -2.777369 | -4.015733 |
| NM_021297 | Tlr4 | toll-like receptor 4 | -4.223671 | -3.583871 | -2.977994 |
| NM_133211 | Tlr7 | toll-like receptor 7 | -6.232589 | -4.388405 | -6.896597 |
| NM_197986 | Tmem140 | transmembrane protein 140 | -3.234025 | -2.927516 | -2.950551 |
| NM_027206 | Tnfaip8l2 | tumor necrosis factor, alpha-induced protein 8-like 2 | -2.937595 | -3.724611 | -4.941386 |
| AK155178 | Tnfrsf13b | tumor necrosis factor receptor 13b | -5.359897 | -4.400903 | -6.28184 |
| NM_022322 | Tnmd | ref\|Mus musculus tenomodulin (Tnmd), mRNA [NM_022322] | -3.498499 | -2.61451 | -3.281681 |
| NM_009405 | Tnni2 | troponin I,2 | -3.363633 | -2.50118 | -3.845409 |
| NM_009421 | Traf1 | Tnf receptor-associated factor 1 | -4.007514 | -3.234337 | -3.255642 |
| NM_031254 | Trem2 | triggering receptor expressed on myeloid cells 2 | -7.515691 | -4.859065 | -8.10547 |
| NM_027763 | Treml1 | triggering receptor expressed on myeloid cells-like 1 | -3.81589 | -2.702044 | -4.050678 |
| NM_133977 | Trf | transferrin | -4.590431 | -2.859539 | -4.47639 |
| BC069182 | Trp73 | transformation related protein 73 | -2.564971 | -3.230526 | -2.788529 |
| NM_012035 | Trpc7 | transient receptor potential cation channel, C7 | -4.009869 | -2.829345 | -4.397856 |
| NM_011662 | Tyrobp | TYRO protein tyrosine kinase binding protein | -7.987015 | -4.98372 | -8.273712 |
| NM_023738 | Ube1l | ubiquitin-activating enzyme E1-like | -3.221057 | -4.491404 | -2.644814 |
| NM_201410 | Ugt1a6b | UDP glucuronosyltransferase 1 A6B | -3.145905 | -2.556779 | -2.502601 |
| NM_019449 | Unc93b1 | unc-93 homolog B1 | -2.514368 | -2.828406 | -3.144204 |
| NM_011691 | Vav1 | vav 1 oncogene | -6.11581 | -3.855033 | -6.434872 |
| NM_028813 | Vit | vitrin | -4.156155 | -4.596035 | -3.145313 |
| NM_011707 | Vtn | vitronectin | -2.730399 | -2.918684 | -3.006857 |
| NM_009515 | Was | Wiskott-Aldrich syndrome homolog | -4.278084 | -3.069502 | -5.067595 |
| AK155186 | Wdfy4 | n/a | -6.04047 | -4.388928 | -6.89138 |
| XM_991190 | Wdr52 | WD repeat domain 52 | -3.142935 | -2.515494 | -2.906212 |
| AK016201 | Wdr69 | G-protein beta WD-40 repeats containing protein | -3.055369 | -2.813326 | -3.005209 |
| NM_027725 | Wdr69 | WD repeat domain 69 | -2.966213 | -2.675617 | -2.765875 |
| NM_016873 | Wisp2 | WNT1 inducible signaling pathway protein 2 | -2.870387 | -3.202071 | -2.523272 |
| NM_011723 | Xdh | xanthine dehydrogenase | -4.271528 | -2.656076 | -4.123704 |
| NM_011725 | Xlr | X-linked lymphocyte-regulated complex | -5.375546 | -3.597193 | -5.04291 |
| NM_025749 | Zfp474 | zinc finger protein | -4.553536 | -3.309148 | -4.984047 |
